# Supplementary figures and images for: Sycp2 is essential for synaptonemal complex assembly, early meiotic recombination and homologous pairing in zebrafish spermatocytes
Source: PLoS Genet. 2020 Feb 24;16(2):e1008640. doi: 10.1371/journal.pgen.1008640 (PMC7062287; doi:10.1371/journal.pgen.1008640)

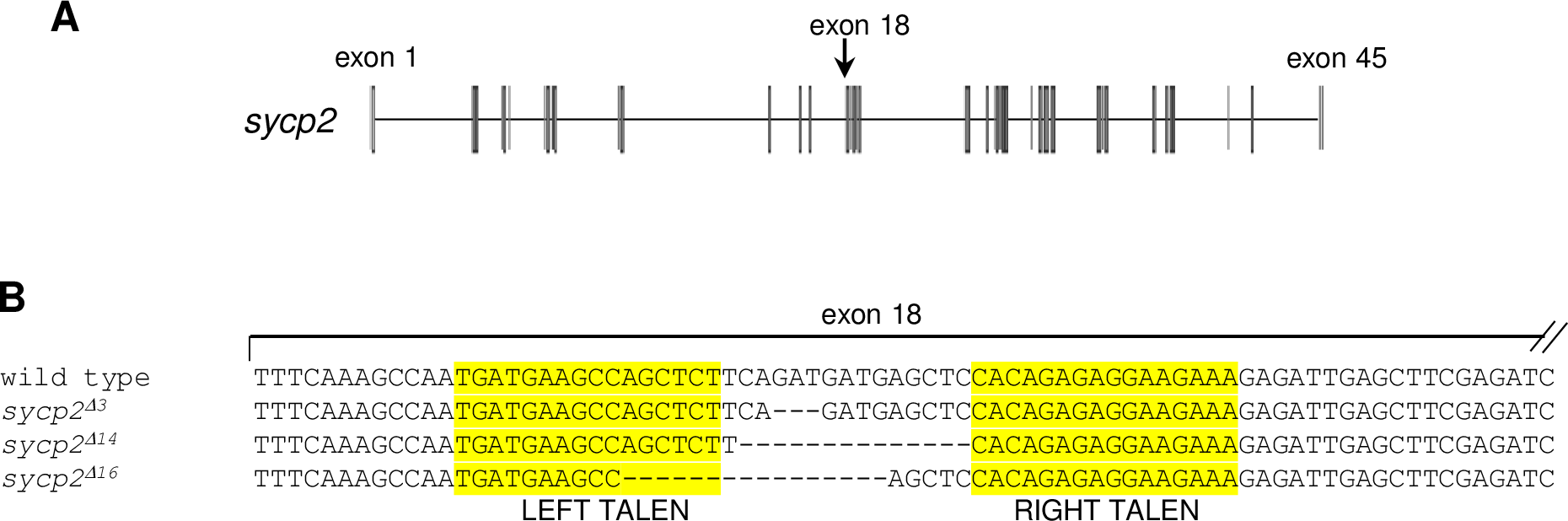

Supplement: S1 Fig — A: A schematic presentation of the exon-intron structure of the sycp2 gene. The Sycp2 protein is coded in 45 exons (shown as vertical lines) that span ~60 kbp on zebrafish chromosome 23. The coding sequences (exons) are based on the annotations for XM_679956.6. B: Mutation sites of sycp2 knockout zebrafish generated by TALEN mutagenesis. The sequences targeted by the TALEN proteins are shown in yellow. Three sycp2 mutant lines with 3-, 14- or 16-bp deletions were isolated. These alleles were named sycp2Δ3, sycp2Δ14 and sycp2Δ16, respectively. (TIF) [file pgen.1008640.s001.tif]

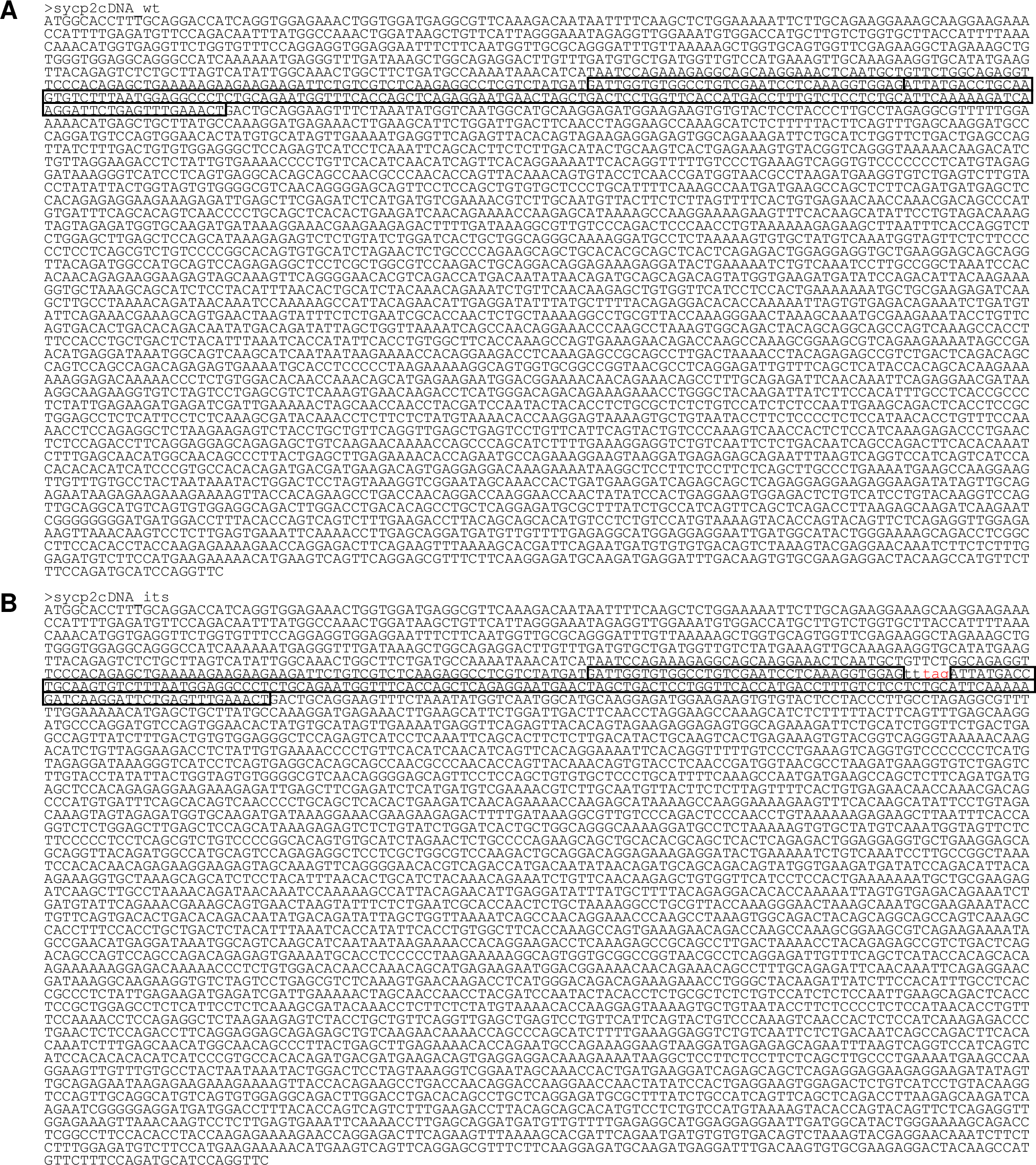

Supplement: S2 Fig — A: A sequence of the full-length wild-type sycp2 cDNA. B: A sequence of the full-length sycp2 cDNA with the insertion of a premature termination codon (in red) by aberrant exon 8–9 splicing. The exon 8 and exon 9 sequences are boxed. Both wild-type (A) and its-type (B) cDNAs were obtained from the same sycp2its/its male fish. (TIF) [file pgen.1008640.s002.tif]

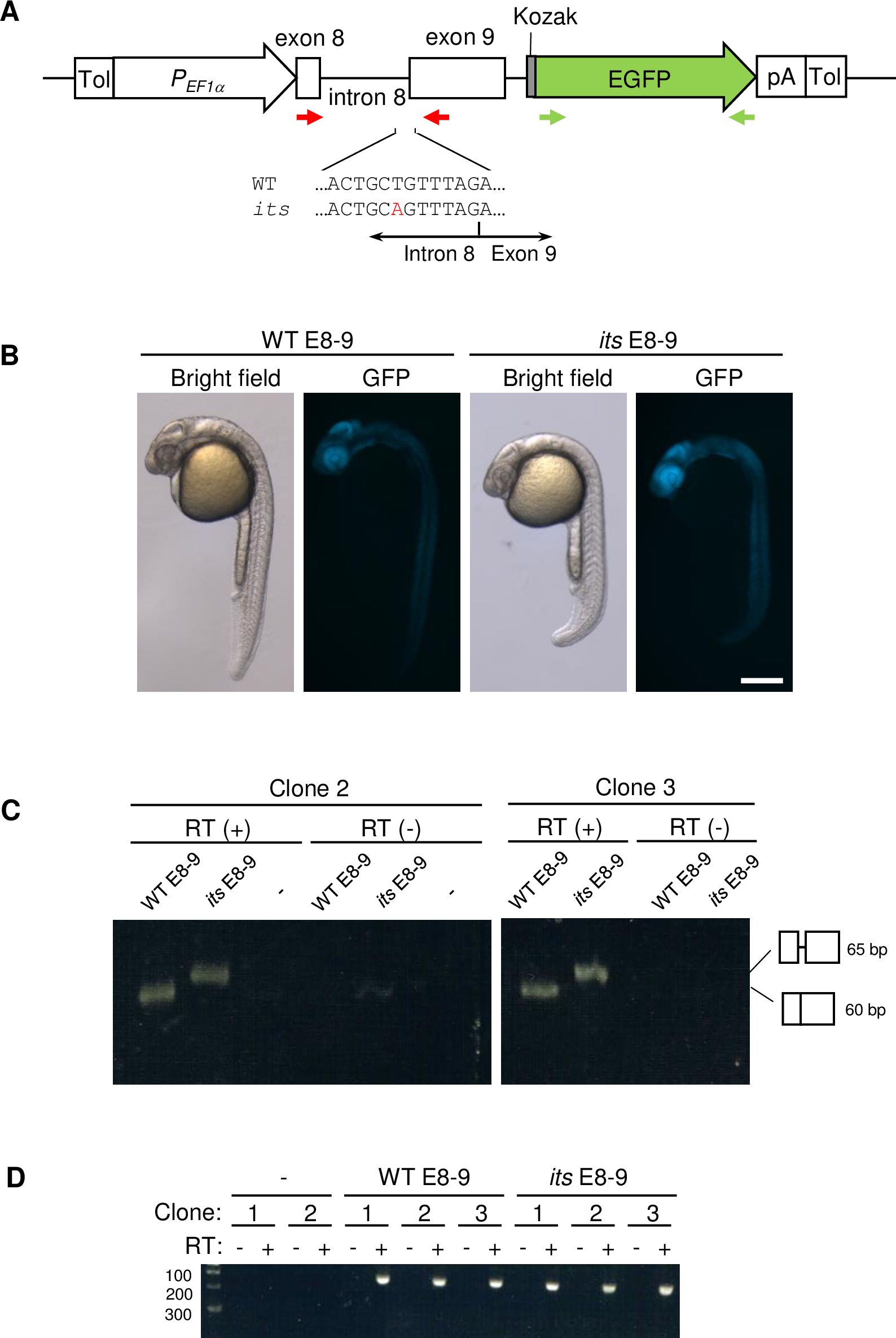

Supplement: S3 Fig — A: A schematic presentation of the mini-gene constructs used in the splicing assay. The mini-genes contained either wild-type (WT) or its-type (its) sequences for sycp2 intron 8 (133 bp) and flanking regions of exon 8 (34 bp) and exon 9 (143 bp). The primers used for RT-PCR to amplify a control GFP sequence (S3 Fig) and to assess exon 8–9 splicing (Fig 2D) are shown as green and red arrows, respectively. Tol, Tol2 transposon sequences. PEF1α, elongation factor 1α promoter derived from Xenopus laevis for ubiquitous expression of the transgene. pA, poly(A) signal. Kozak, Kozak consensus sequence. The size of each element does not correspond to its actual sequence length. B: Expression of GFP in transgenic embryos with wild-type (WT E8-9) and its-type (its E8-9) mini-genes. Bright-field and fluorescent (GFP) images of embryos at one day postfertilization. The fluorescent signals indicate mini-gene expression. Scale bar, 300 μm. C: Mini-gene splicing assay in the wild-type genetic background. In addition to the clones shown in Fig 2D, RT-PCR was performed with two more transgenic fish each with either wild-type (WT E8-9) or its-type (its E8-9) sycp2 mini-genes. One more wild-type fish without mini-genes was used as a control (-). D: Expression of the GFP reporter gene in transgenic fish with mini-genes. RT-PCR was performed with caudal fin cDNA from two individual wild-type fish without a transgene (-) and three individual transgenic fish with either wild-type (WT E8-9) or its-type (its E8-9) sycp2 mini-genes. The clones labeled “1” and those labeled “2” and “3” were used for RT-PCR in Fig 2D and S3 Fig, respectively. Non-RT (-) controls were subjected to PCR using total RNA processed without an RT reaction. (TIF) [file pgen.1008640.s003.tif]

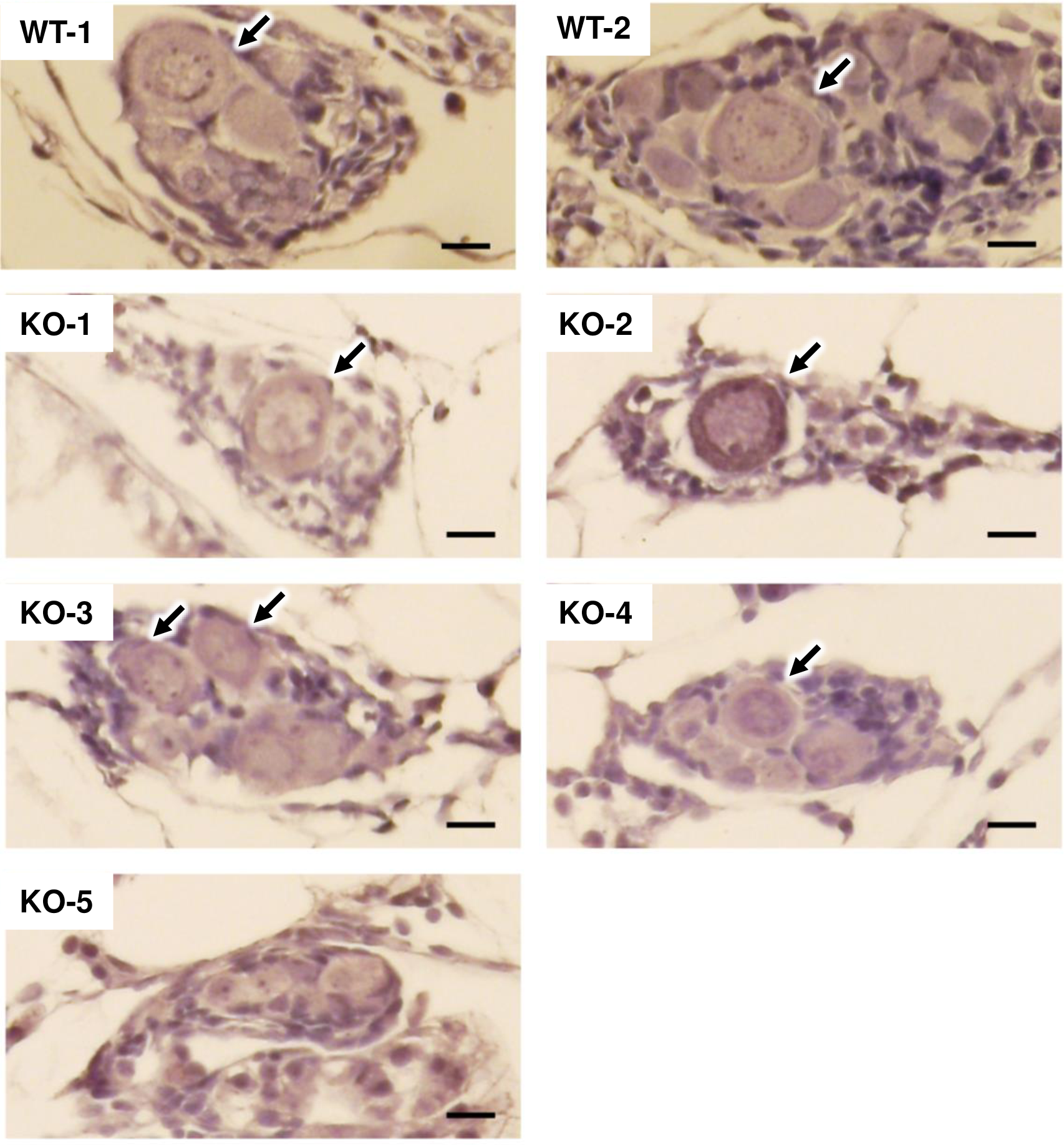

Supplement: S4 Fig — HE-stained gonads of two sycp2+/+ (WT-1 and -2) and five sycp2-/- (KO-1 to -5) zebrafish at 28 days postfertilization. Arrows indicate late stage IB oocytes [36]. Scale bars, 20 μm. (TIF) [file pgen.1008640.s004.tif]

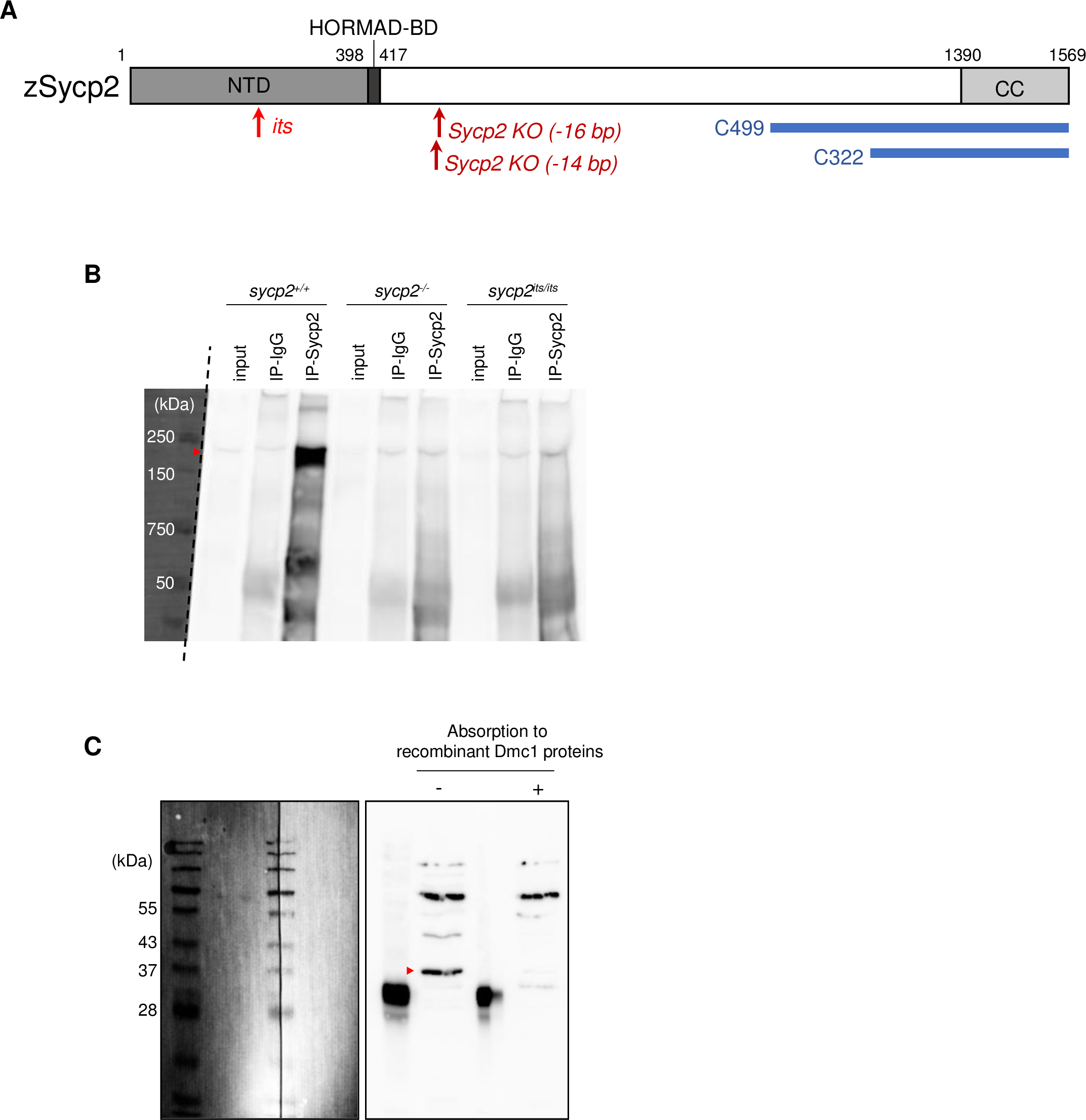

Supplement: S5 Fig — A: A schematic model of the zebrafish Sycp2 protein. The full-length structure of the 1569-amino-acid sequence is shown, with regions similar to mammalian SYCP2 domains: NTD, N-terminal domain; HORMA-BD, putative HORMA-binding domain; CC, C-terminal coiled-coil domain [19]. The C-terminal regions used as immunogens to generate anti-Sycp2 antibodies (C499 and C322) are indicated as blue bars. The positions of premature stop codon in sycp2 mutant lines are indicated with red arrows. B: Western blotting of sycp2+/+, sycp2-/- and sycp2its/its testis protein extracts using an anti-Sycp2 antibody. Immunoprecipitation was performed with protein extracts from sycp2+/+, sycp2-/- and sycp2its/its testes using a guinea pig anti-Sycp2 antibody (IP-Sycp2) or normal guinea pig IgG as a control (IP-IgG). SDS-PAGE was performed on 7.5% TGX Precast Gel (Bio-Rad). Each well was loaded with an immunoprecipitated sample or 0.4% input. Immunoblotting was performed with a rat anti-Sycp2 antibody. The predicted size of Sycp2 is 176 kDa. The left part is a colorimetric image of the protein ladder on the same membrane. The Sycp2 protein was not detected in sycp2its/its testes. However, we cannot exclude the possibility that there is expression of a truncated Sycp2 protein that is not recognized by our anti-Sycp2 antibodies specific to a C-terminal region of Sycp2. C: Western blotting of wild-type testis extract with anti-Dmc1 guinea pig antiserum. Each well was loaded with 28 μg of protein. The predicted size of zebrafish Dmc1 is 38 kDa. After blocking in TBST with 5% skimmed milk, the membrane was incubated with the anti-Dmc1 guinea pig antiserum at a 1:2500 dilution and with a biotinylated anti-guinea pig antibody at 1:1000; then, the signals were amplified with a VECTASTAIN ABC kit (Vector Labs) and developed with an ECL Plus kit (lane -). Blotting was also performed with anti-Dmc1 antiserum after absorption to recombinant Dmc1 proteins (lane +) as a control. The left image is a colorime [file pgen.1008640.s005.tif]

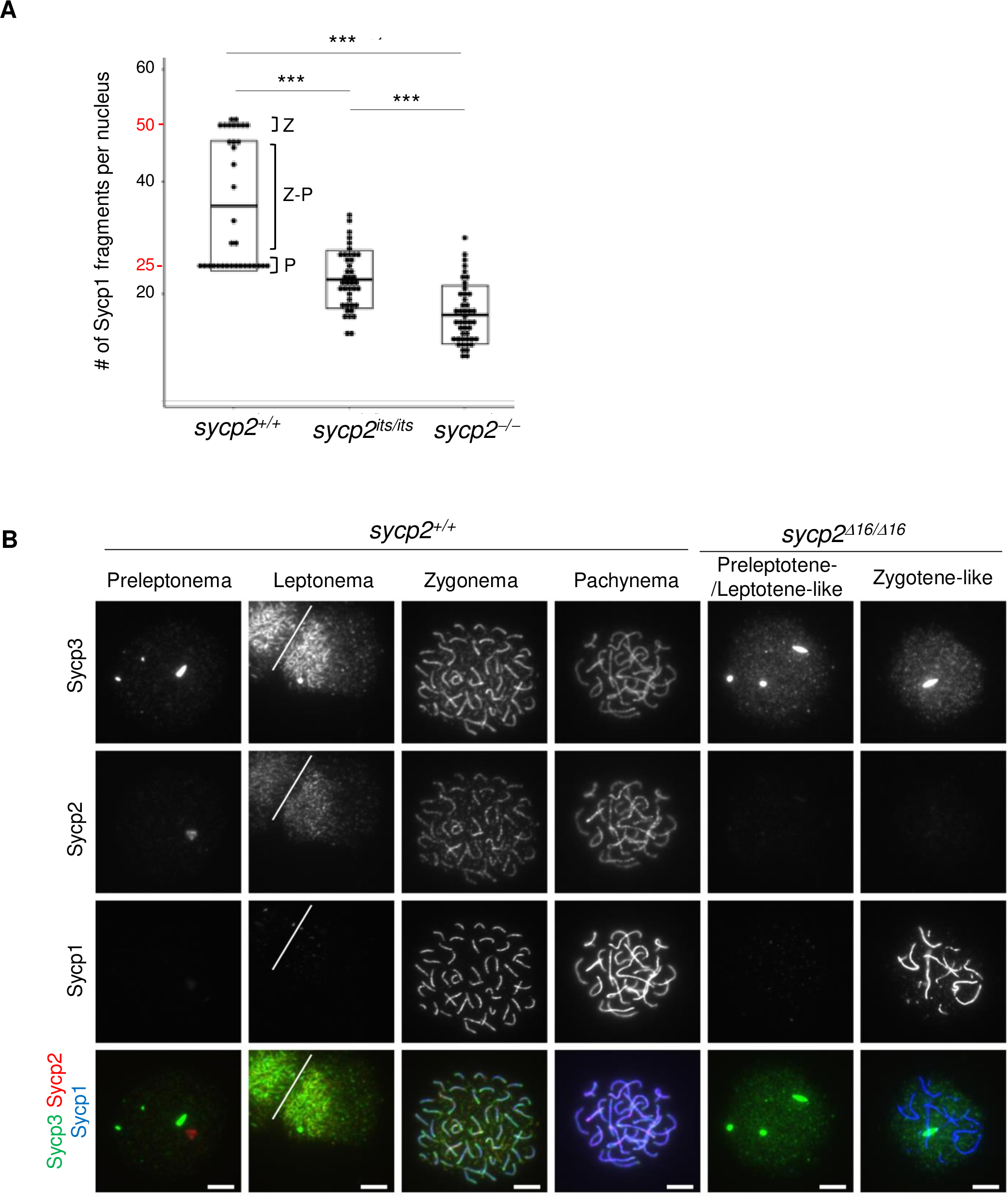

Supplement: S6 Fig — A: Quantification of the number of Sycp1 fragments per nucleus in sycp2+/+, sycp2its/its and sycp2-/- (sycp2Δ14/Δ14) spermatocytes. Quantification was performed for nuclei stained with an anti-Sycp1 antibody, a telomere-targeting polyamide and DAPI (see Materials and Methods). Since zebrafish have 25 pairs of homologous chromosomes with telomeres at both ends, nuclei containing ~50 and 25 Sycp1 fragments are at zygonema and pachynema, respectively (Z and P). Wild-type nuclei containing between 25 and 50 Sycp1 fragments are likely at the zygotene-pachytene transition (Z-P). The boxes indicate the SD, with the mean value as the middle bar. Chromosomal spreads of one (wild-type) or two (sycp2its/its and sycp2-/-) individual fish were used for counting. sycp2+/+, n = 34; sycp2its/its, n = 41; and sycp2-/-, n = 45. *** indicates p<0.0001 (Student’s t-test). B: Immunostaining of SC components on sycp2+/+ and sycp2Δ16/Δ16 spermatocyte chromosomal spreads. Individual images with anti-Sycp3, anti-Sycp2, or anti-Sycp1 antibodies and a merged image are shown for each nucleus. The white line on the wild-type leptotene image indicates a nuclear border with another nucleus on the top left. Scale bars, 5 μm. (TIF) [file pgen.1008640.s006.tif]

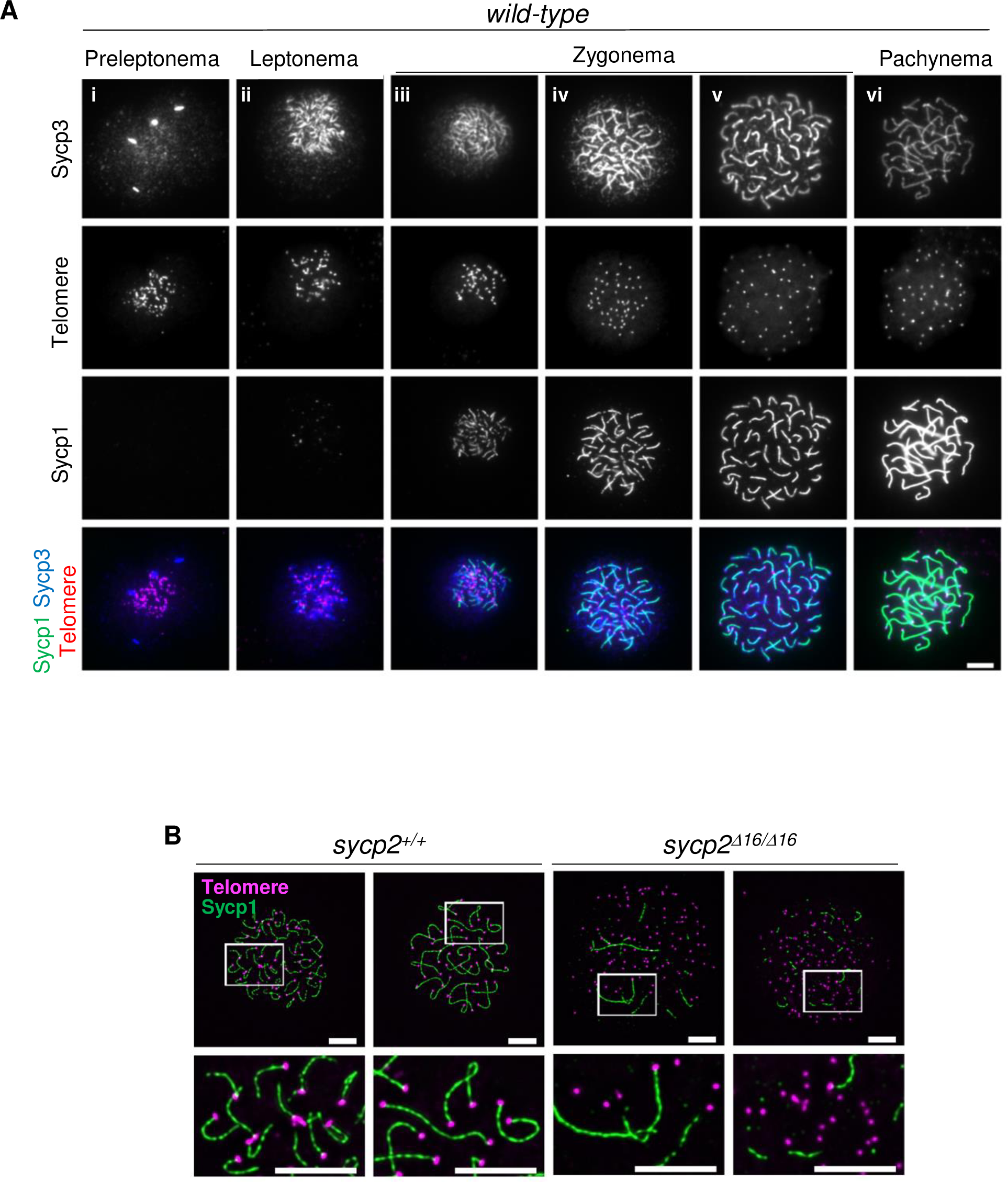

Supplement: S7 Fig — A. Individual images with telomere-targeting polyamide, anti-Sycp3 antibodies or anti-Sycp1 antibodies and a merged image are shown for each nucleus. The stages of the nuclei were determined based on the Sycp3 and Sycp1 signals, according to a recent report by Blokhina et al. [26]: in preleptonema, telomeres cluster together to form a bouquet (i); this step is followed by the extension of Sycp3 filaments from the clustered telomeres at leptonema (ii); in zygonema, following the inward extension of the Sycp3 filaments, Sycp1 exclusively emanates from telomeres that disperse throughout the nuclei, resulting in the dismantling of the telomere bouquet; Sycp1 and Sycp3 filaments extend along the entire lengths of chromosomes emanating from both telomeres in pachynema (vi). B: Costaining of telomeres and Sycp1 on sycp2+/+ and sycp2Δ16/Δ16 spermatocyte chromosomal spreads. The regions outlined in white are shown at a higher magnification at the bottom. The nuclei of sycp2+/+ spermatocytes are at zygonema (left; telomeres detected at one end of each Sycp1 filament) and pachynema (right; telomeres detected at both ends of Sycp1 filaments). Scale bars, 5 μm. (TIF) [file pgen.1008640.s007.tif]

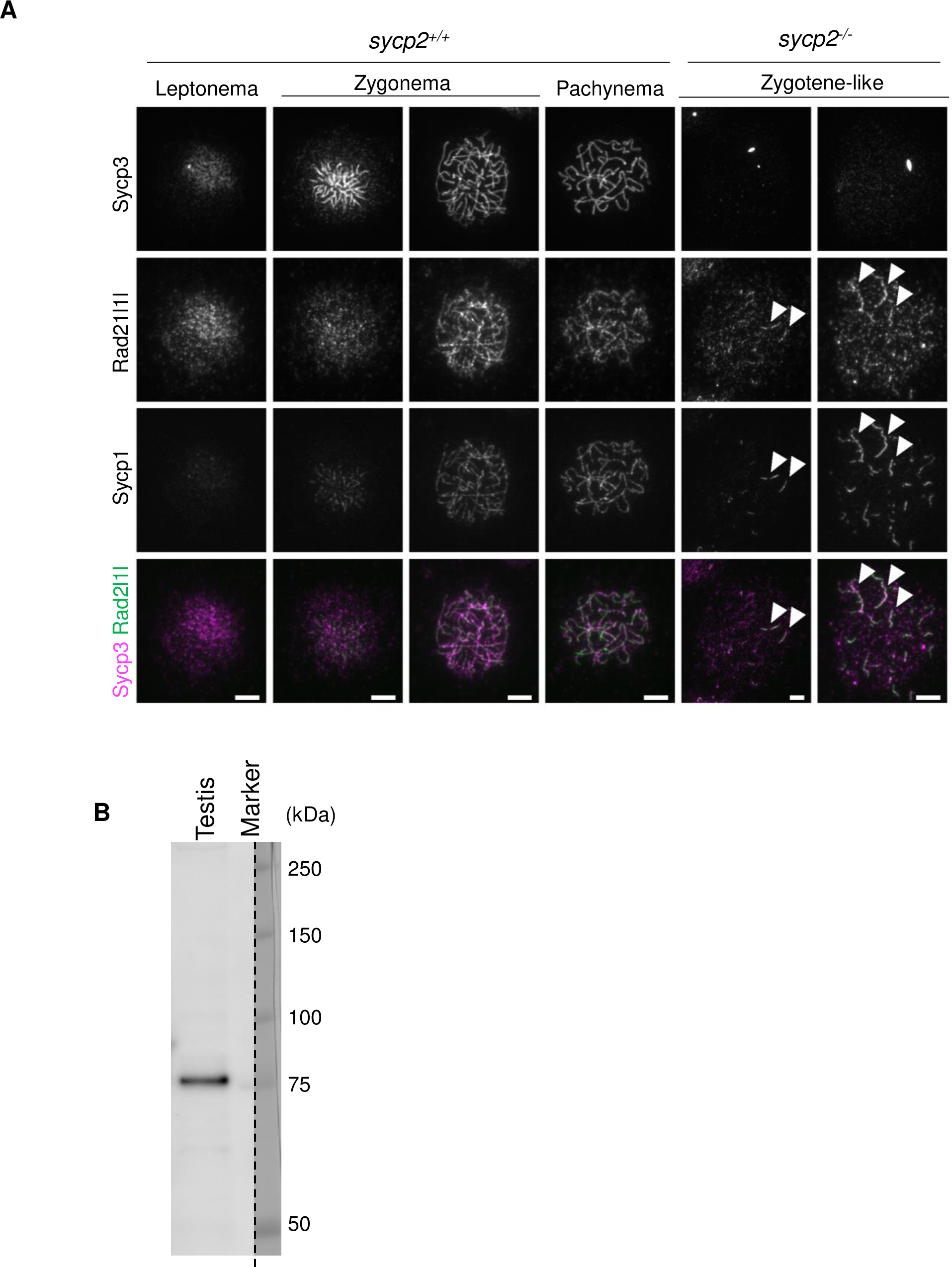

Supplement: S8 Fig — A: The meiotic cohesin Rad21-like 1 (Rad21l1) was costained with Sycp1 and Sycp3. In wild-type spermatocytes, Rad21l1 showed similar localization to Sycp3 throughout meiotic prophase I, although scattered signals were also observed over nuclei. In sycp2-/- spermatocytes, Rad21l1 was observed as mottled stretches on Sycp1 filaments as well as scattered signals over nuclei. Arrowheads indicate Rad21l1 costained with Sycp1 filaments in sycp2-/- spermatocytes. Scale bars, 5 μm. B: Western blot of zebrafish testis extract with the anti-Rad21l1 antiserum used in S8A Fig. Adult testis proteins were extracted in RIPA buffer, and 50 μg of protein was migrated on a 10% SuperCep acrylamide gel (Wako). After blocking in TBST with 5% skimmed milk, the membrane was incubated with the anti-Rad21l1 mouse antiserum at a 1:150 dilution and with anti-mouse IgG HRP-conjugated antibody at 1:2000; then, the signals were detected with an ECL Prime kit. The right part of the image is a colorimetric image of the protein ladder on the same membrane. Although a single band appeared larger than the predicted molecular weight of zebrafish Rad21l1 (63 kDa), such difference has been observed for mouse RAD21L [67]. (TIF) [file pgen.1008640.s008.tif]

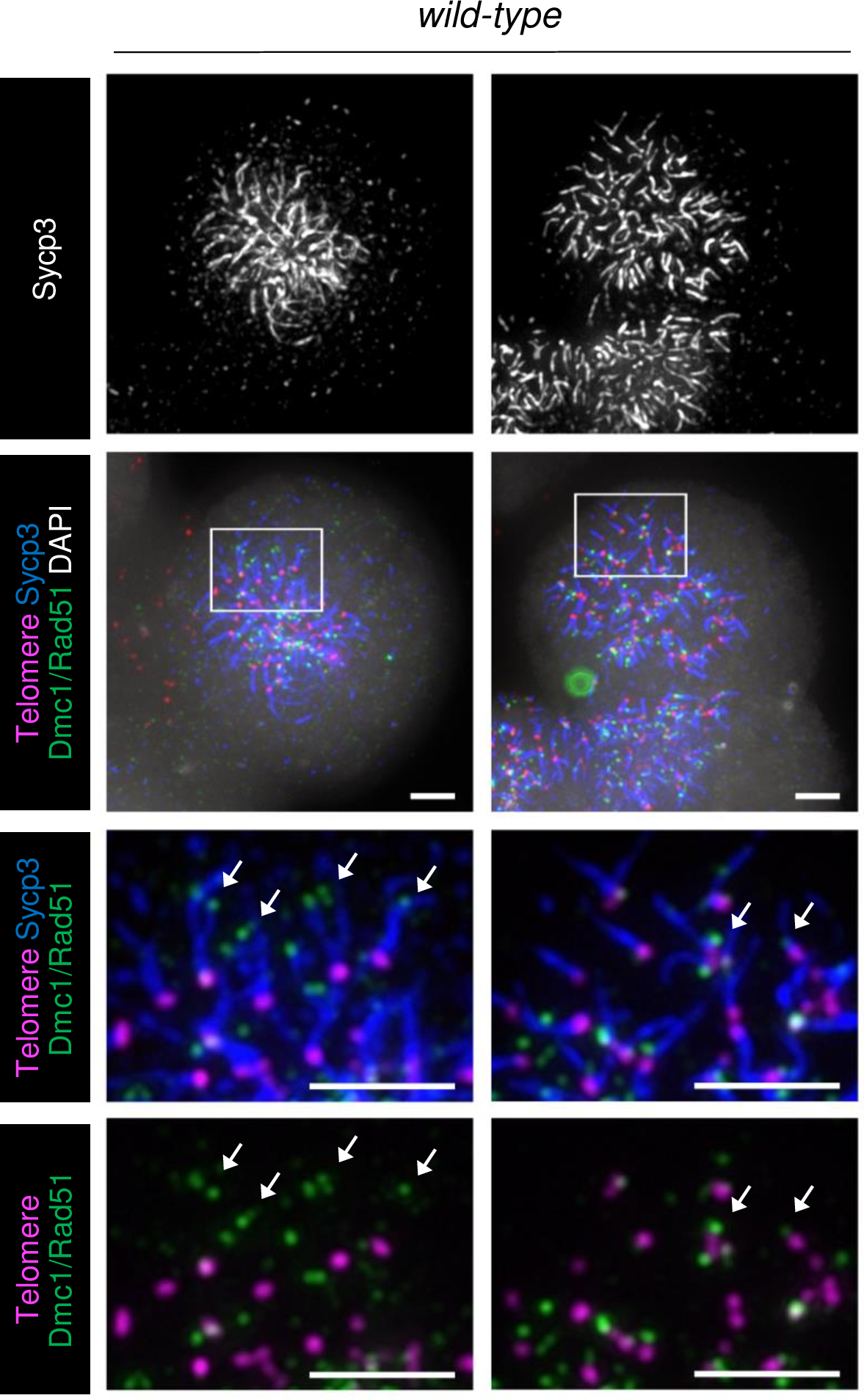

Supplement: S9 Fig — Individual images with anti-Sycp3 antibody and merged images are shown for two wild-type nuclei at leptonema to early zygonema. The merged images show staining for Sycp3 (blue), telomeres (magenta), Dmc1/Rad51 (green) and DAPI (white). The regions enclosed by white rectangles are shown in a higher magnification with or without Sycp3 (images in the second and third rows). Scale bars, 5 μm. (TIF) [file pgen.1008640.s009.tif]

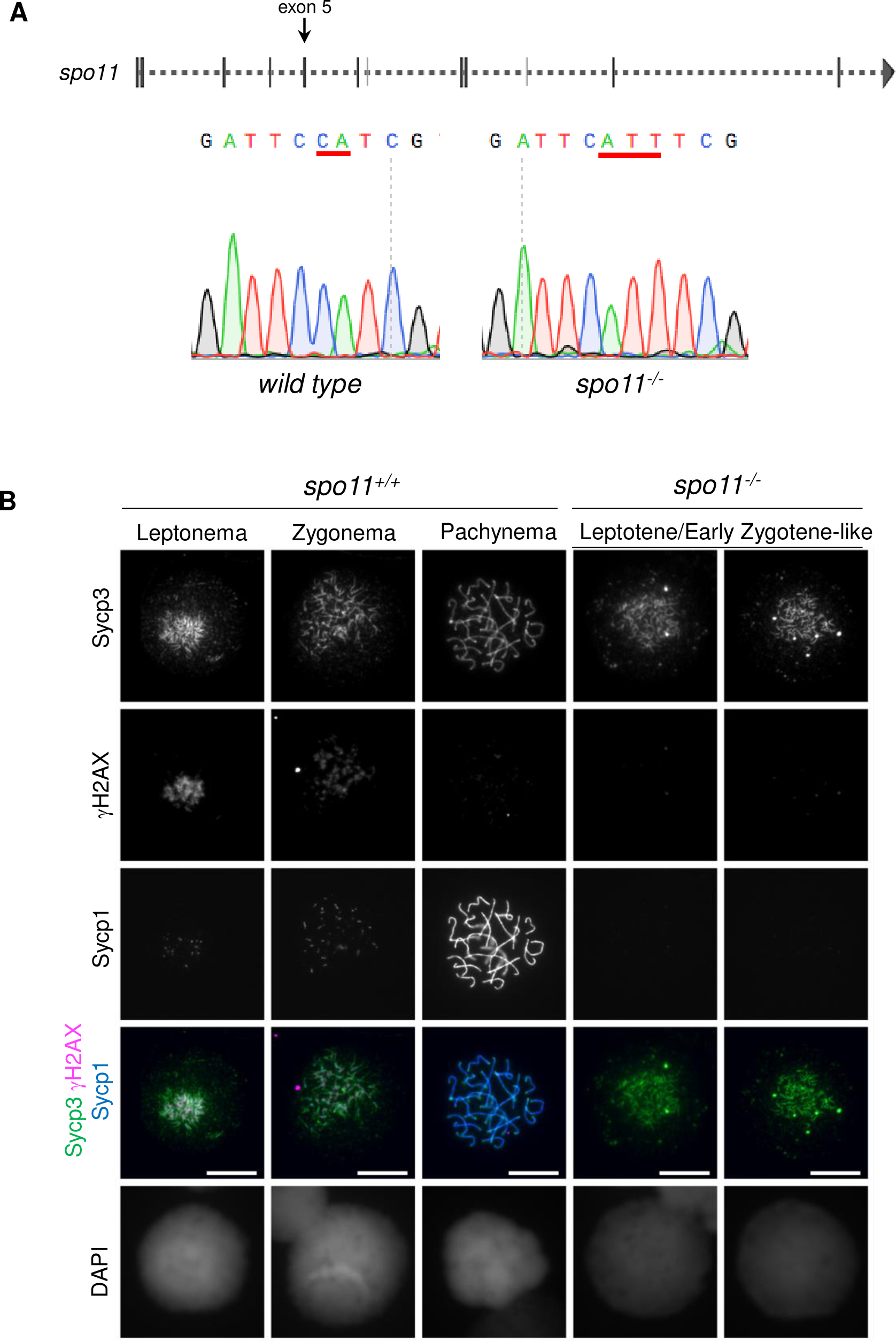

Supplement: S10 Fig — A: A schematic presentation of the exon-intron structure of the spo11 gene. We isolated a mutant with a +1 frameshift caused by substitution of "CA" with "ATT" in exon 5. In this study, this mutation is referred to as the spo11- allele. The sequencing data for the mutation site in spo11+/+ (wild-type) and spo11-/- are shown. B: Staining of γH2AX on spo11+/+ and spo11-/- spermatocyte chromosomal spreads. In wild-type spermatocytes, γH2AX signals were detected at leptonema to early zygonema, as we reported previously [25]. In contrast, γH2AX signals were rarely detected in spo11-/- spermatocytes with Sycp3 patterns similar to those in wild-type leptonema or early zygonema. Thus, there were no detectable DSBs in our spo11-/- zebrafish spermatocytes as determined by γH2AX staining. Scale bars, 5 μm. (TIF) [file pgen.1008640.s010.tif]

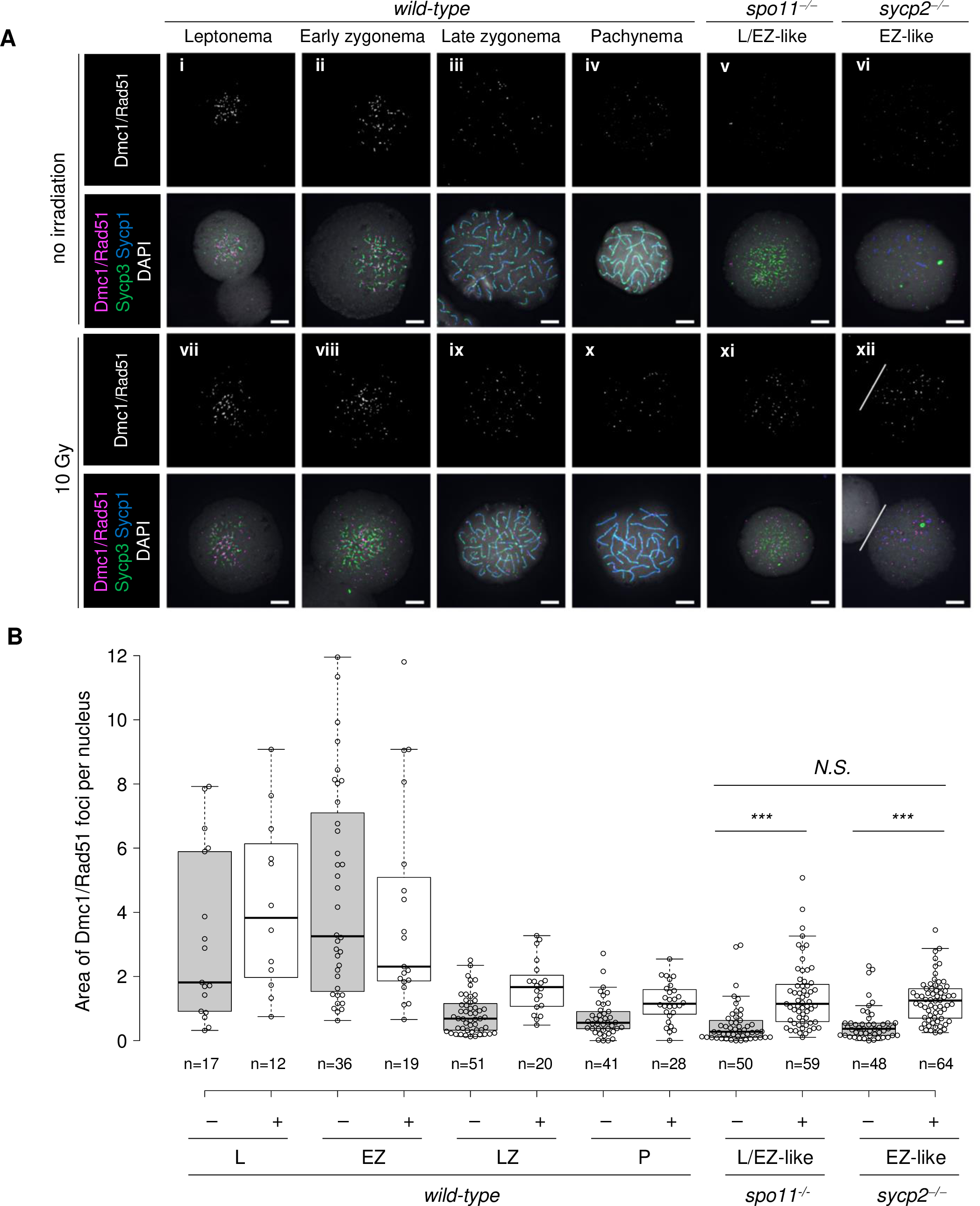

Supplement: S11 Fig — A: Immunostaining of Dmc1/Rad51, Sycp1 and Sycp3 on non-irradiated (no irradiation; i to vi) and γ-ray irradiated (10Gy; vii to xii) spermatocyte chromosomal spreads of wild-type (i to iv, vii to x), spo11-/- (v and xi) and sycp2-/- (vi and xii). The wild-type nuclei are at leptonema (i and vii), early zygonema (ii and viii), late zygonema (iii and ix) and pachynema (iv and x) according to the Sycp1 and Sycp3 staining patterns. spo11-/- nuclei at a leptotene- or early zygotene-like stage (L/EZ-like), according to Sycp3 staining patterns, are shown (v and xi). Early zygotene-like (EZ-like) sycp2-/- nuclei stained with short Sycp1 fragments are shown (vi and xii). The white line on the irradiated sycp2-/- image (xii) indicates a nuclear border with another nucleus on the top left. Scale bars, 5 μm. B: Quantification of the Dmc1/Rad51-stained area in non-irradiated (-) and γ-ray irradiated (+) spermatocytes. The sum of the area stained for the Dmc1/Rad51 foci in each nucleus was measured in wild-type nuclei at leptonema (L), early zygonema (EZ), late zygonema (LZ), and pachynema (P), in leptotene- or early zygotene-like (L/EZ-like) spo11-/-, and in early zygotene-like (EZ-like) sycp2-/- spermatocytes. Numbers at the bottom (n) indicate numbers of nucleus measured in each data set. Center lines show the medians; box limits indicate the 25th and 75th percentiles as determined by R software; whiskers extend 1.5 times the interquartile range from the 25th and 75th percentiles; data points are plotted as open circles. Chromosomal spreads of four (wild-type) and two (spo11-/- and sycp2-/-) individual fish were used for each condition. *** indicates p<0.0001 (Student’s t-test). N.S. indicates not significant. (TIF) [file pgen.1008640.s011.tif]

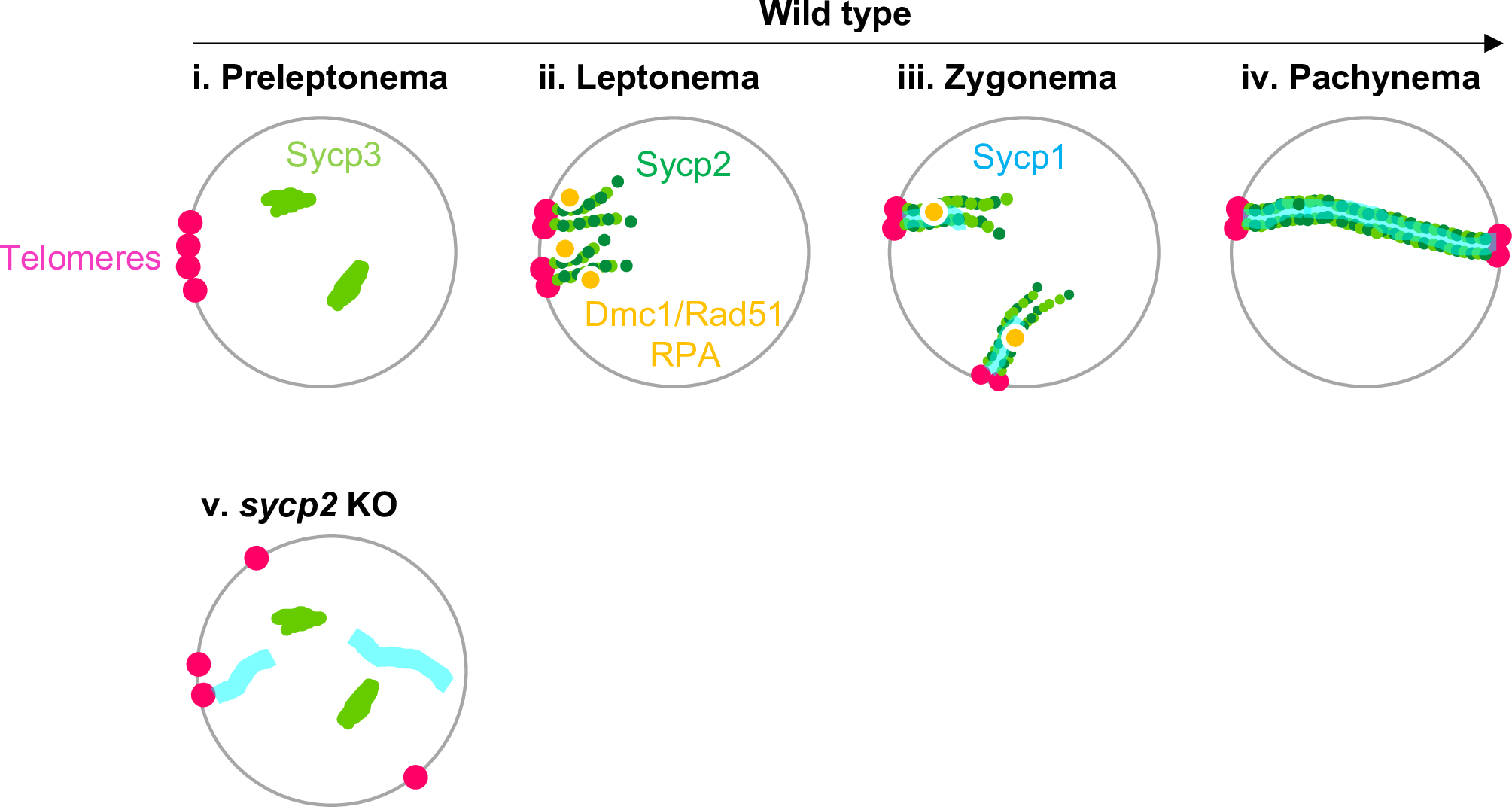

Supplement: S12 Fig — In zebrafish spermatocytes, Sycp3 is expressed in aggregates before leptonema (i; preleptonema), and telomeres are observed in bouquets. In leptonema, upon axis formation of Sycp2 near telomeres, Sycp3 is also localized on an axis (ii). In the same stage, DSB formation also occurs in the proximity of the telomeres, as indicated by Rad51 [26], Dmc1/Rad51 and RPA staining. In zygonema, synapsis is also initiated near telomeres by the localization of Sycp1 (iii). Synapsis is completed at pachynema, and telomeres are dissociated from bouquets at this time (iv) [26]. When we knocked out Sycp2, the signals of DSB markers were rarely detected, and homologous pairing was strongly impaired (v). (TIF) [file pgen.1008640.s012.tif]

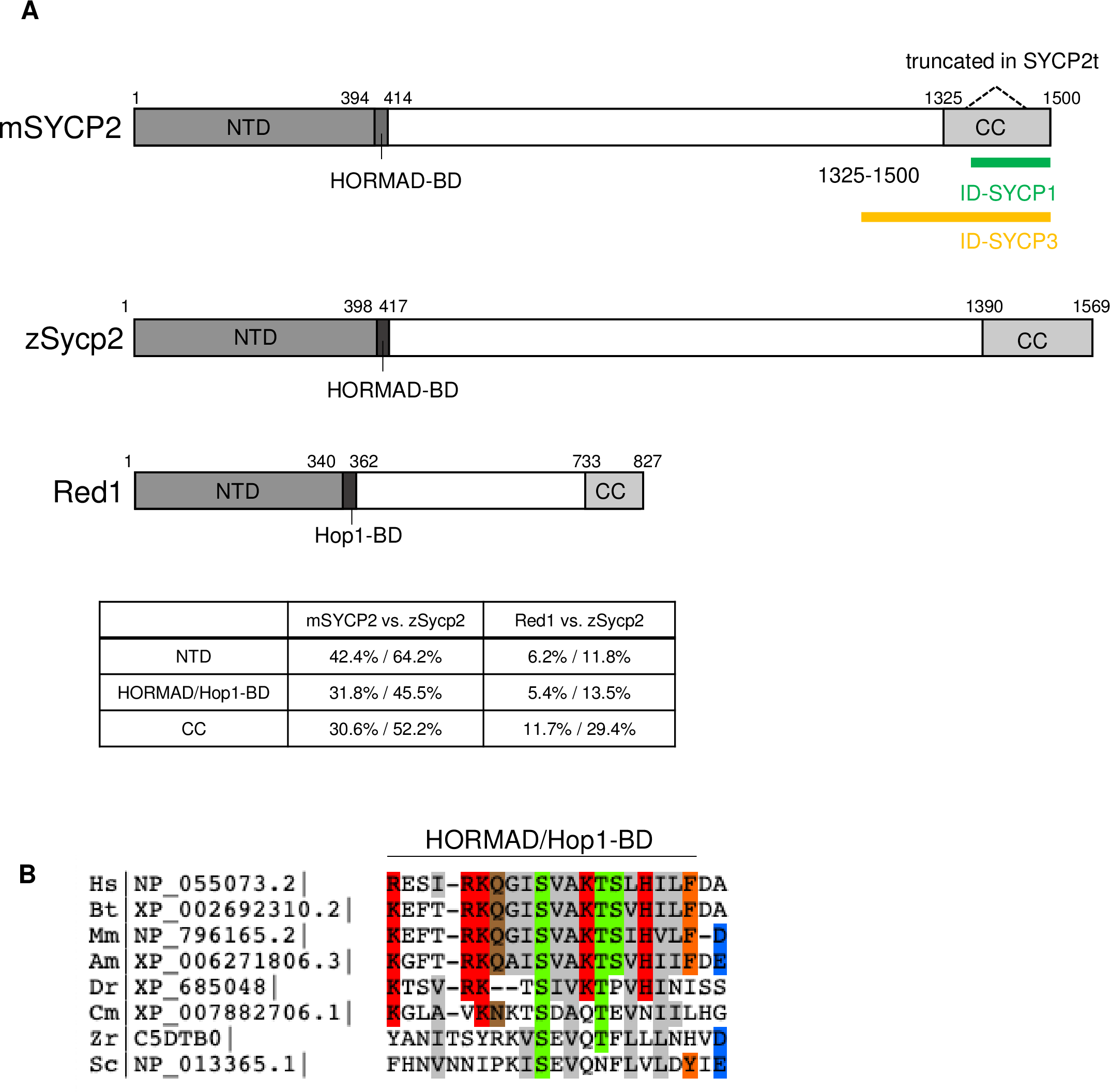

Supplement: S13 Fig — A: Schematic presentations of domain structures of mouse SYCP2 (mSYCP2), zebrafish Sycp2 (zSycp2) and budding yeast Red1. Domain structures of mSYCP2 and Red1 were from [19]. NTD, N-terminal domain; HORMA/Hop1-BD, putative HORMA/Hop1-binding domain; CC, C-terminal coiled-coil domain. The numbers in the panel correspond to the positions of the amino acid residue. C-terminal regions of mSYCP2 that are involved in interactions with SYCP1 (ID-SYCP1) [23] and SYCP3 (ID-SYCP3) [22] are shown in green and yellow bars, respectively. A C-terminal region truncated in the previously published Sycp2 mutant mouse (SYCP2t) is also shown [22]. Percent identity/similarity between each domain of zSycp2 and that of mSYCP2/Red1 is shown at the bottom. B: The alignment of HORMA/Hop1-binding domains (HORMA/Hop1-BD) across species. Alignment was performed with sequences of putative HORMA/Hop1-BDs from Homo sapiens (Hs), Mus musculus (Mm), Alligator mississippiensis (Am), Danio rerio (Dr), Callorhinchus milii (Cm), Zygosaccharomyces rouxii (Zr), and Saccharomyces cerevisiae (Sc) by the T-coffee program. Conserved residues with similar properties were visualized by the MView program. Reference IDs of each sequence are indicated in the panel. (TIF) [file pgen.1008640.s013.tif]

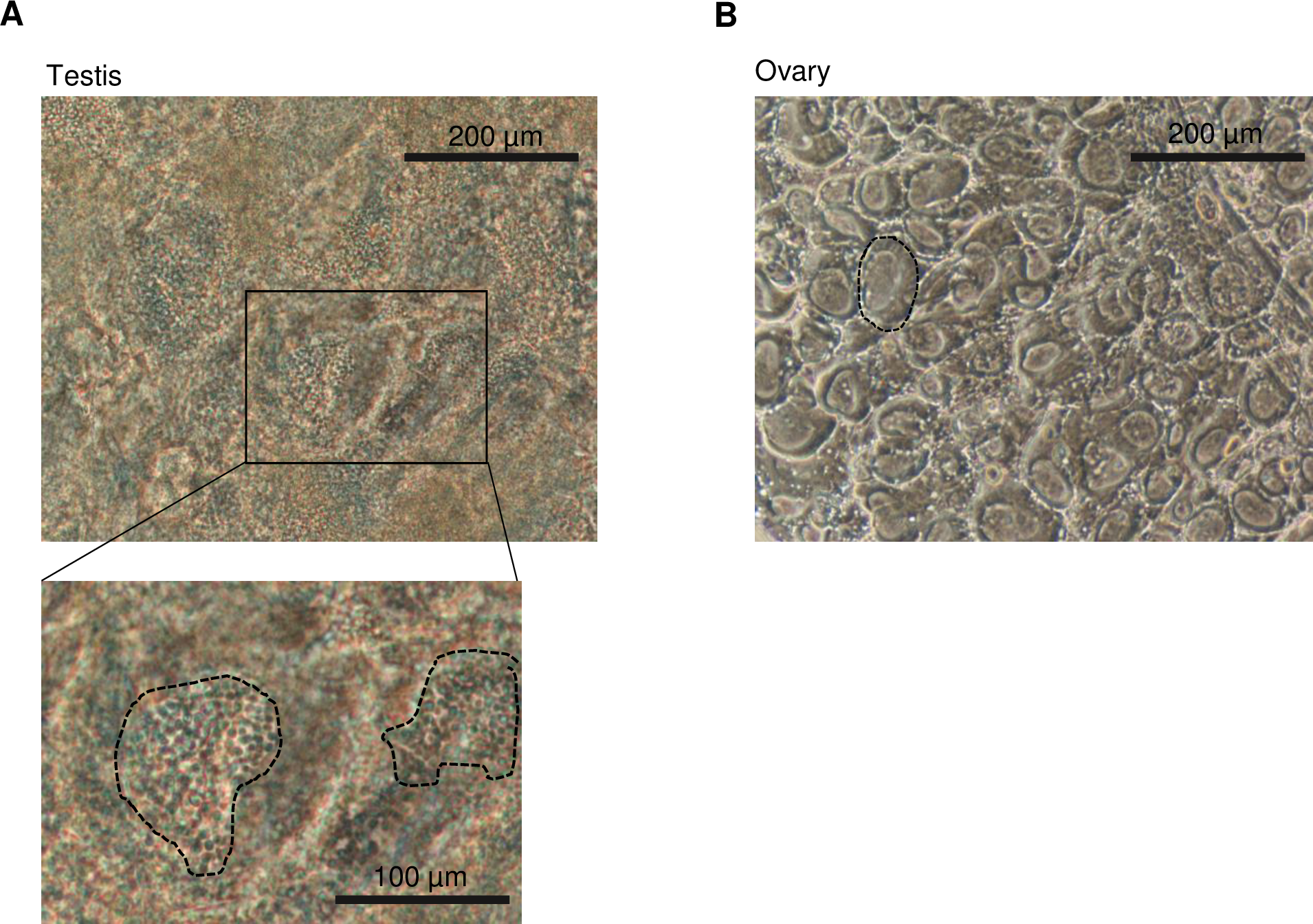

Supplement: S14 Fig — A: A light field image of zebrafish testis. The region outlined with black is shown at a higher magnification at the bottom. Spermatids and/or sperm cells are outlined with broken lines. B: A light field image of zebrafish ovary with an oocyte outlined in broken lines. (TIF) [file pgen.1008640.s014.tif]

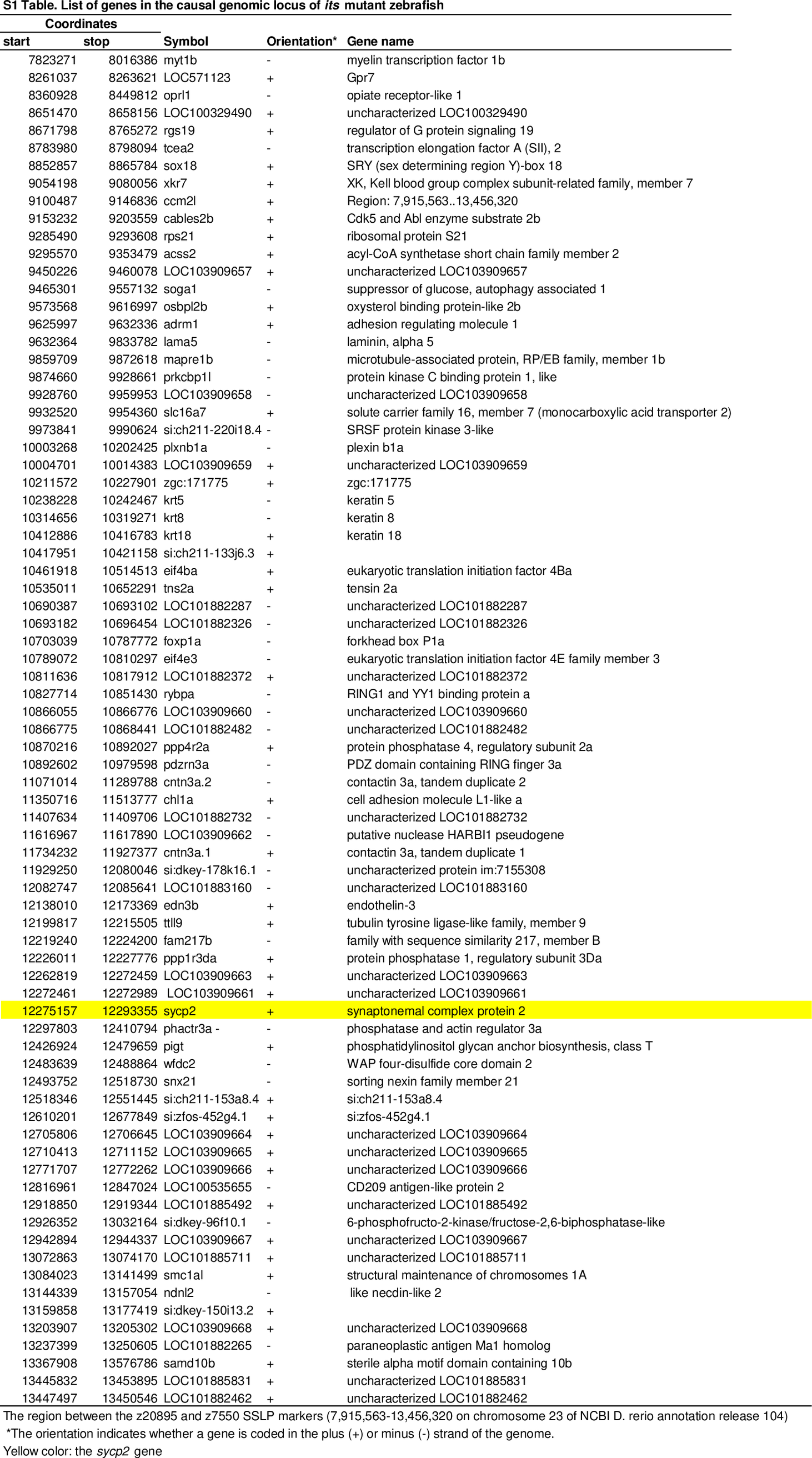

Supplement: S1 Table — (TIF) [file pgen.1008640.s015.tif]

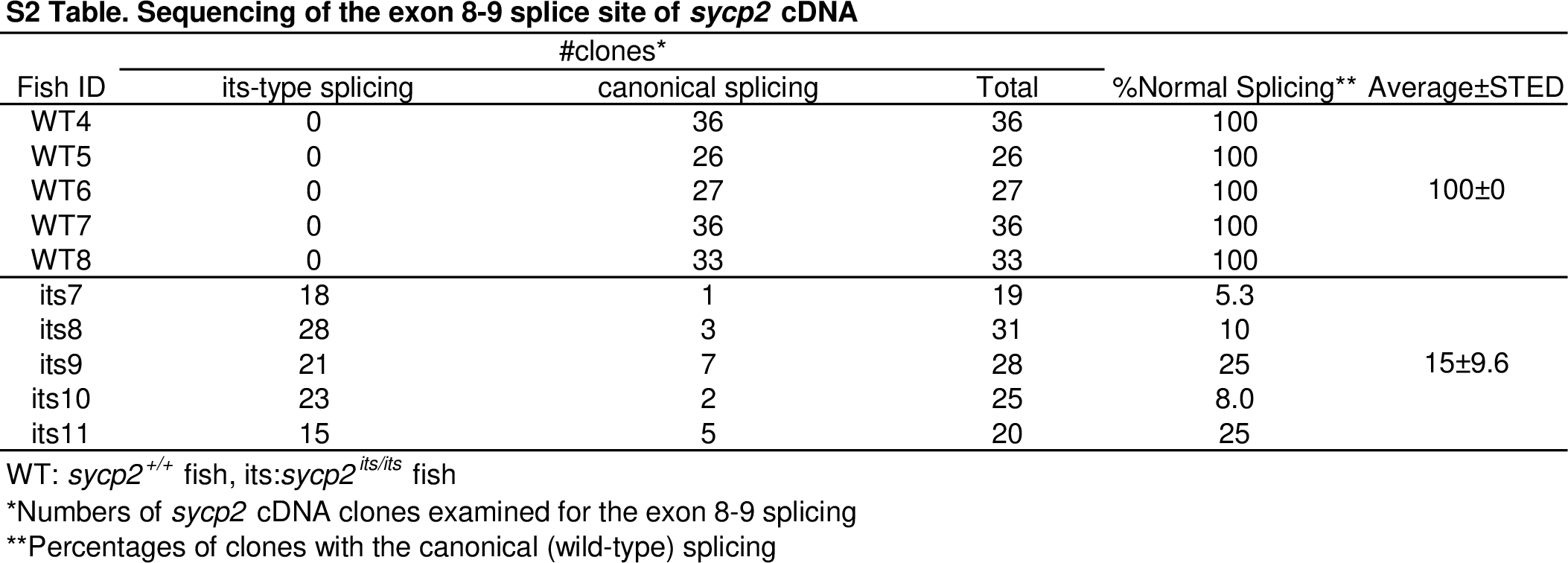

Supplement: S2 Table — (TIF) [file pgen.1008640.s016.tif]

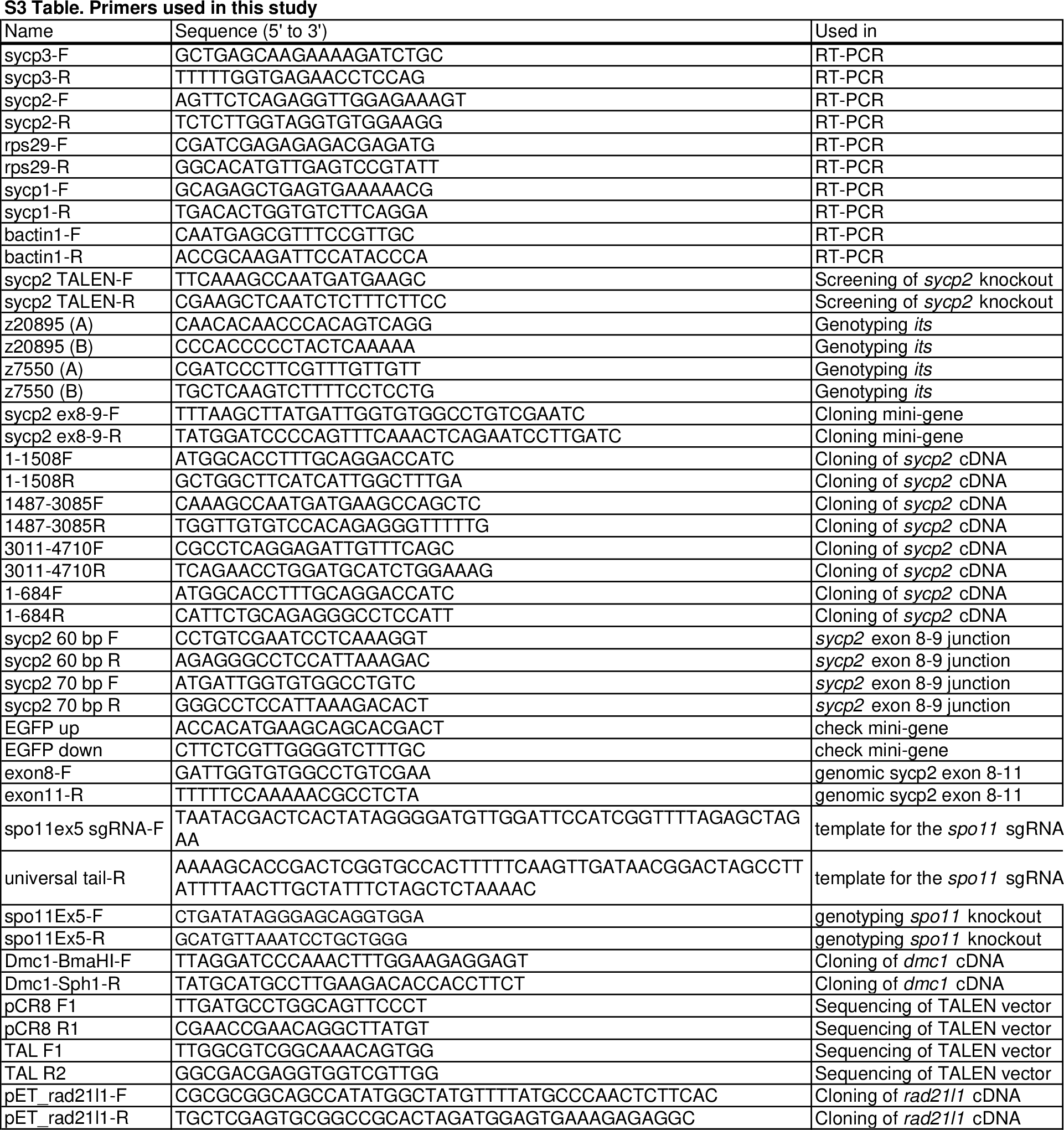

Supplement: S3 Table — (TIF) [file pgen.1008640.s017.tif]

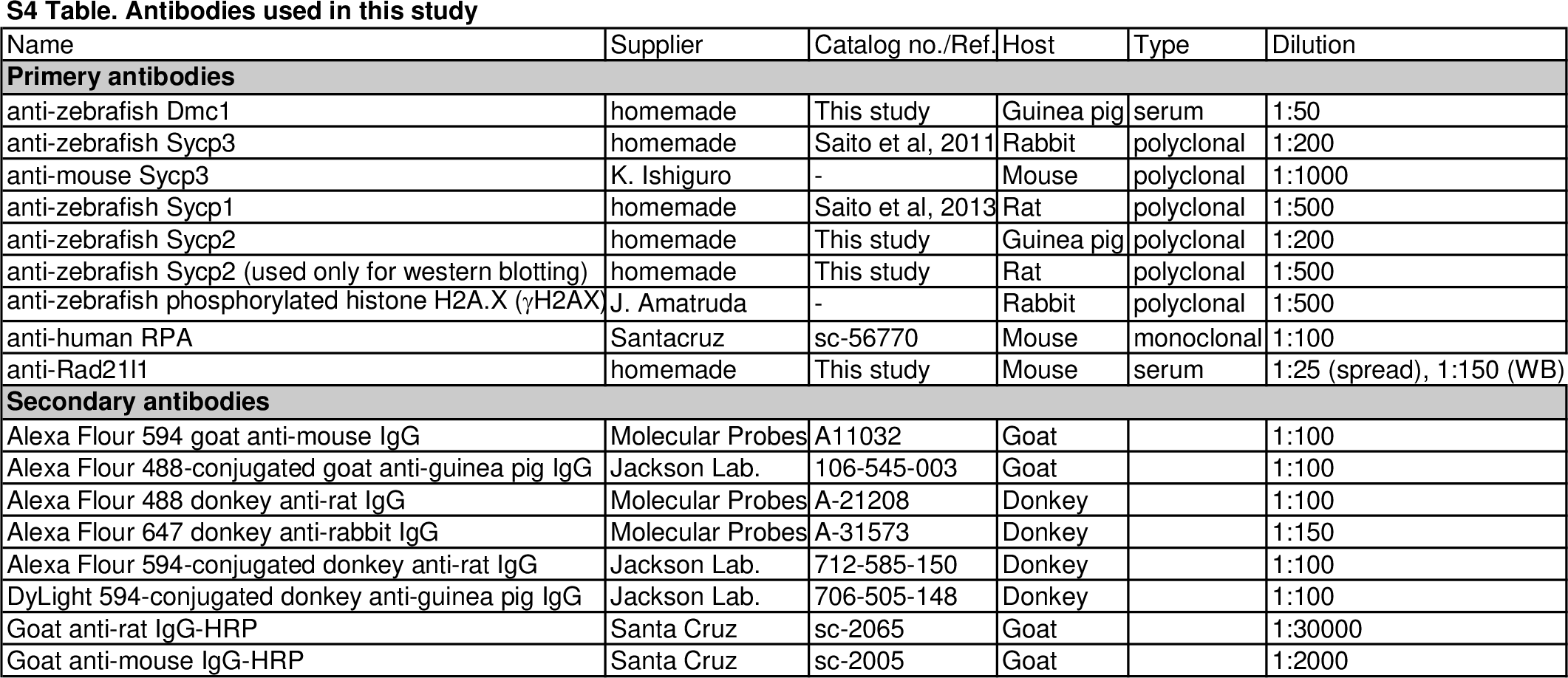

Supplement: S4 Table — (TIF) [file pgen.1008640.s018.tif]
