# Supplementary material for: Sycp2 is essential for synaptonemal complex assembly, early meiotic recombination and homologous pairing in zebrafish spermatocytes
Source: PLoS Genet. 2020 Feb 24;16(2):e1008640. doi: 10.1371/journal.pgen.1008640 (PMC7062287; doi:10.1371/journal.pgen.1008640)
Supplement: Attachment — Submitted filename: Comments_to_the_Authors_191102.docx [file pgen.1008640.s020.docx]

Reviewer #1: The the zebrafish Ietsugu (its) mutant was previously identified by the authors in a forward genetic screen for male sterility mutants and was found to be defective in generating the meiotic chromosome axis. In this study, the researchers map the its mutation to the sycp2 locus and show it is a T to A substitution that causes aberrant splice site usage. The its mutation behaves as a hypomorphic allele with expression levels ~26% of wild type. The authors created a TALEN generated knockout of the sycp2 gene and find it is required for axis formation (i.e. Sycp3 staining) and DSB formation (Dmc1 staining), but that some SC (Sycp1) forms ectopically. The zebrafish sycp2-/- mutant phenotypes are comparable to a SYCP2-/- mouse. Several new resources were developed including antibodies raised to zfDmc1 and zfSycp2 that could be useful for the burgeoning zebrafish meiosis field.

The work describing the sycp2 phenotypes is solid and the immunofluorescence figures are very nice and support the novel conclusions of the paper. In addition, having a hypomorphic allele in hand adds to the study. As zebrafish is an emerging model for meiosis, understanding the evolutionary conservation of function of potential orthologs is important so identifying the its mutation as an allele of Scyp2 is significant.

There are several issues that need to be addressed before publication. Several conclusions made by the authors are either not supported by the data or are not based on novel observations (e.g. Sycp2 as a potential functional ortholog of yeast Red1, DSBs occur near telomeres in zebrafish, zebrafish as a model for human male meiosis). The comments below suggest ways that the paper can be better focused to highlight the novel aspects of the work.

Comments:
1. The authors suggest that they have discovered Sycp2 as a functional ortholog of yeast Red1 protein since pairing, DSB formation, axis formation and SC assembly are defective in the sycp2-/- mutant. Reporting these data and discussing its relevance to Red1 is important, but arguing that they have made the novel discovery that Sycp2 is the functional Red1 ortholog is a bit of an overstatement. The link between Red1 and Sycp2 has been explored previously in mouse studies and recent structural analysis has revealed commonalities and differences between the two proteins (West, eLIFE 2019; Feng, 2107). Red1 protein forms a homotetramer while mouse SYCP2/SYCP3 forms an analogous heterotetrameric structure. One of the main arguments the authors make supporting the notion that Scyp2 is a potential ortholog of Red1 stems from the failure to see DSBs in the zebrafish sycp2 mutant and previous reports that DSBs form in the mouse SYCP3-/- mutant. This is rather circumstantial evidence. To really nail this down, the authors would need to create the zebrafish sycp3-/- mutant which seems beyond the scope of the current paper. The authors should also be aware that yeast red1 mutant does in fact form DSBs, but they are repaired with the sister chromatid (Blat et al, 2002). Most of the discussion on pages 16 and 17 could be cut so that the authors say briefly that their findings are consistent with the notion that Scyp2 is the functional ortholog of Red1.

­­­––– We appreciate this comment and rewrite our phrases saying that Sycp2 is a potential counterpart of Red1. Accordingly, we rewrote the last sentence in introduction and shortened the corresponding part of the last discussion section because we did not generate *sycp3* mutant zebrafish in this study. This section was merged with the second to the last section about DSB formation near telomeres, according to the 4th comment of this reviewer and a suggestion from another reviewer.

2. The authors suggest that Sycp2 is required for ovarian development in zebrafish based on a sex reversal phenotype described in a previous publication (pages 15-16). Granted, other mutations affecting oogenesis have been shown to cause sex reversal, but these studies have also shown that sex reversal is suppressed by tp53-/- mutation, further suggesting a defect in meiotic chromosome dynamics during oogenesis. To make the claim that sex reversal is due to a check point arrest, the scyp2-/- tp53-/- double mutant should be made.

––– We agree that analyzing the *sycp2^-/-^ tp53^-/-^* double mutant fish is very important to conclude that our sex reversal phenotype in *sycp2^-/-^* fish is caused by a checkpoint mechanism. However, we did not have time to generate this mutant fish since it takes at least a half year.

In zebrafish, since individuals in which these immature oocytes degenerate become males, mutants depleted of oocytes develop as males that are mostly. Therefore, the sex reversal phenotypes of zebrafish *sycp2^-/-^* suggest the depletion of oocytes by a certain stage that is required for ovarian development. This seems to be a severe phenotype compared with mice [Yang et al. J Cell Biol 2006]. In addition, we observed gonads of *sycp2* mutants at 28 days postfertilization which contained late stage IB oocytes (S10 Fig). Oocytes of *sycp2* mutants seem to develop slightly later than those of *fancl* and *brca2* mutants [Rodriguez-Mari et al. PLOS Genet 2011, Shive et al. PNAS 2011]. Accordingly, we should analyze the checkpoints and the elimination mechanisms of the oocyte carefully. The study requires much time and should be addressed in our future experiments. We have removed the sentence in the discussion claiming that sex reversal might be controlled by a checkpoint arrest and added the above discussion.

3. In addition, there should be some documentation on how many fish were analyzed for a sex reversal phenotype and the criteria used for sexing fish. Without directly scoring gonads with arrested oocytes, it is a bit of an overstatement to conclude that Scyp2 is required for ovarian development. It would be important to know if some small fraction of females escape sex reversal. Perhaps females arise more frequently in the its mutant? If so then analysis of ovarian development could be carried out on those females. If the tp53-/- mutation suppresses the sex reversal phenotype then there should be a population of females where ovarian development could be analyzed. Exploring this phenotype in more detail would be interesting since the phenotype is markedly different from the spo11 mutant, which also fails to pair or synapsis chromosomes, but produces fertile oocytes.

––– To confirm the sex reversal phenotype of *sycp2* knockout zebrafish and to compare with *spo11* knockout phenotypes, we performed an intercrossing of each line and evaluated their sex and genotypes using the same criteria, as newly added in the 3rd result section. These results are shown in Table 1.

We did not perform intercrossing of the *its* line because of space and time limitations. However, we have not observed females among the homozygous mutant fish since we started the genotyping of the *its* line using linked SSLP markers in our laboratory. We agree that analyzing the ovarian phenotypes of the i*ts^-/-^ tp53^-/-^* and *sycp2^-/-^ tp53^-/-^* double mutant females is very interesting to understand the elimination mechanism of oocytes; however, we could not generate these mutant fish because of time limitations. We would like to address this point in future work.

4. The authors also elaborate on their observations that DSBs form near telomeres in males (pages 14-15). This is not a novel observation and has been reported in other zebrafish studies, including a paper by the author (Saito et al, Dev Dyn 2011, 2014; Sansam and Pezza 2015 (not cited) and Blokhina et al, 2019). This section should be shortened to focus the discussion on the role of Sycp2 in DSB formation.

­­­––– According to this comment, we referred to the previous papers and shorten the text. Accordingly, we moved Fig4A (telomere staining in wild-type spermatocytes) in the previous manuscript to the supplementary data (S6 Fig) in the new manuscript.

5. The formation of SC stretches in the absence an axis is interesting. Perhaps the authors could speculate how the SC is forming without an axis. Is there a precedence for this? Could they be forming between sister chromatids?

––– To clarify this point, we generated an antiserum against the meiotic cohesin Rad21l1. Costaining of Rad21ll and Sycp1 in *sycp2* knockout spermatocytes was added to S7 Fig. We observed the localization of Rad21l1 on Sycp1 stretches in *sycp2* knockout spermatocytes. Therefore, the cohesin axis seems to exist even in the absence of Sycp2, and Sycp1 stretches could be formed between sister chromatids.

Minor points:

Line 173: “Sycp1 began to be observed as short fragments followed by the appearance of Sycp3 and Sycp2 signals”. Is this correct? The figure looks like Sycp1 forms fragments after Scyp3 and Scyp2.

–––– The words "followed by" were a mistake of "following". We corrected the sentence.

Reviewer #2: The Manuscript of Takemoto et al presents the characterization of SYCP2 in zebrafish through the cloning of the its locus as a splice-site mutation in the sycp2 gene. The creation of additional alleles with similar phenotypes and the development of spreading techniques allow the authors to demonstrate defects in pairing, synapsis, and DSB formation/early repair intermediate processing and the interdependencies with SYCP3 and SYCP1.
The experiments are clearly presented and well controlled and the elucidation of SYCP2 functions is novel. A number of the details of wild type meiosis and the characterization of pairing at telomeres are partially redundant with the recent work from Sean Burgess’ lab, although some of the approaches are complementary rather than redundant.

1. Overall, the paper could benefit from a bit of reorganization. Since SYCP2 is an axis proteins, it seems that it would make sense to present the role of mutation in pairing and then synapsis and DSB formation.

­­­­­­––– Since Sycp2 is a component of the synaptonemal complex, we first focused on phenotypes of other SC components in the absence of Sycp2. Therefore, we kept the original organization.

2. Given the Burgess paper has recently described staging of the events of meiosis with SYCP3 staining and telomere probes, it would behoove the authors to spend less time on the naming of the stages rather than the differences seen in the sycp2 mutants.

––– We removed a part of text naming stages in the 3rd result paragraph and a large part of the 4th result paragraph describing telomere staining from the main text. Accordingly, Fig 4A (costaining of telomeres, Sycp3 and Sycp1 in wild-type spermatocytes) in the previous manuscript was rearranged to S6 Fig A in the new manuscript.

3. In the discussion of sex reversal, the authors should consider the possibility that the SYCP2 and may activate checkpoints exclusively in the female that eliminate oocytes (and female development), allowing for male development. Differences in meiotic checkpoint activation is a feature of numerous species.

––– We speculate that the checkpoint is rather active in both oogenesis and spermatogenesis in *sycp2^-/-^* zebrafish. In zebrafish, all juveniles develop gonads with immature oocytes regardless of their definitive sex. The individual in which these immature oocytes degenerate becomes male. Therefore, zebrafish mutants depleted of oocytes develop as males that are mostly infertile. In *sycp2^-/-^* juvenile gonads, we observed previtellogenic dictyate oocytes in four among five individual fish (S10 Fig). Therefore, the absence of females in *sycp2* mutant fish (Table 1) implies that oocytes were eliminated at the juvenile age, possibly due to checkpoint activation in oogenesis. As a result, all *sycp2* mutant fish develop as males. In adult *sycp2* mutant males, spermatogenesis is arrested at zygotene-like stages as we observed (Fig 3B), suggesting that a checkpoint mechanism could also exist in males. These phenotypes are slightly different from the mouse *Sycp2^-/-^* mutant, which shows a less severe phenotype in females than in males [Yang et al. J Cell Biol 2006]. We should carefully examine whether these checkpoints in *sycp2* mutant females and males share the same mechanism. The study requires much time and should be addressed in our future experiments. We added this discussion to the new manuscript.

4. Related to this, the difference between spo11 and sycp2 mutants vis-à-vis sex reversal suggests either 1) that DSBs are made in sycp2 but are incapable of recruiting DMC1 and H2AX (this could potentially be testing by irradiating the fish, as is often down in worm meiosis to show DSB rescue) or 2) that a synapsis checkpoint is killing off female cells. Given the uniqueness of these phenotypes, it would be valuable to add these details to the main text and perhaps examine checkpoints in the fish, if possible. Indeed, no mention is made of a female phenotype even in the weak loss of functions. Is there one?

­­­––– (1) To rescue DSBs, we performed γ-ray irradiation of *sycp2^-/-^* and the control *spo11^-/-^* fish at doses that have been used to introduce DSBs in *C. elegans* and mouse meiocytes (5 Gy and 10 Gy). However, we could not observe a significant increase in Dmc1 foci in irradiated spermatocytes or recovery of fertility after mating irradiated males with wild-type females in both mutant fish. Thus, we could not find a good experimental condition in the given time. We also tried to generate an anti-Spo11 antibody to detect Spo11-oligo complexes in *sycp2^-/-^* zebrafish. However, we could not obtain any antibody that specifically recognized Spo11. The study requires much time.

––– (2) As confirmed in Table 1, *sycp2^-/-^* homozygote fish do not appear among females, though we observed previtellogenic dictyate oocytes in four of the five *sycp2* mutant juveniles (S10 Fig). Thus, the *sycp2* mutant females seem to have severe phenotypes than *spo11* mutant females. However, it is difficult to discuss a synapsis checkpoint that kills off oocytes. We have to analyze further mutant juveniles in detail, or produce double mutant such as with *tp53* knockout, that can possibly suppress oocyte loss caused in *sycp2^-/-^* oocytes. Both studies require much time. We would like to address these questions in a future work.

Minor comments:
In leptonema in Fig. 3A, it is unclear what the upper right corner is. Is this a different cell? A zoom in of a part of the image?

––– The upper right corner of the leptotene image in Fig. 3A-ii is another nucleus. We added an explanation for the white line separating these nuclei in the figure legend.

Line 173, it is unclear what is meant by “followed by” since they are fixed images and the stains are superimposable.

––– That was a mistake in the use of "following", since Sycp1 fragments appear after Sycp3 and Sycp2 signals. We corrected this sentence.

For continuity, in Figure 4, SYCP3 should be on top and SYCP1 below (similar to figure 3).

––– We rearranged the panels in S6 Fig A (Figure 4A in the previous manuscript).

It would be helpful to label Figure 4A as wild type.

––– We added a label of wild-type to S6 Fig A (Figure 4A in the previous manuscript).

Figure S6 B should indicate in labels colocalization with telomere probes.

––– We modified the axis label of Fig 4B (Figure S6 B in the previous manuscript) according to this comment.

Reviewer #3: The synaptonemal complex is a meiosis-specific chromosome structure that is critical for the execution of recombination during gamete development. While previous studies in mammals have focused on the effects of chromosome axis component Sycp2 on synaptonemal complex assembly and synapsis, its role in DSB formation has not yet been investigated in mammals. As evidenced by recent papers (including Blokhina et al PLoS Genetics 2019), the use of zebrafish as a model system for studying meiosis has proven powerful for understanding meiotic processes in organisms that initiate SC formation in subtelomeric regions (e.g., humans). In the following manuscript by Takemoto et al., the authors have identified an allele of the conserved synaptonemal complex protein Sycp2 through a previously published forward genetic screen for zebrafish sterility phenotypes (Saito et al., Developmental Dynamics 2011). The authors convincingly demonstrate that this allele acts as a hypomorph of variable penetrance arising from a splicing defect due to a mutation within exon 8 of sycp2. Further, the authors utilized TALEN mutagenesis to generate a null allele of sycp2 and characterized its meiotic phenotypes. Using the combination of these two alleles in zebrafish, the authors clearly show that Sycp2 is required for sperm production in males. The lack of sperm production appears to be due to defects that occur during meiotic prophase I: assembly of the synaptonemal complex, pairing of homologs, and meiotic DSB formation. Based on these phenotypes and the fact Sycp2 is a meiotic chromosome structure component, the authors provide support for the conclusion from West et al., eLife 2019 that Sycp2 serves as an axis component analogous to the well-studied Red1 in S. cerevisiae and HTP-3 in C. elegans. Further, similar to spo11 and other meiotic null mutations, the authors find that loss of sycp2 prevents the development of viable female zebrafish.　Overall, the evidence that the authors provide for the role of Sycp2 in assembly of the SC and DSB formation is convincing. Taken together, the authors provide a good characterization of two alleles of a known meiotic chromosome axis protein homolog.

Comments to improve the conclusions and impact of the manuscript:

1. The authors qualitatively show a decrease in meiotic DNA damage by immunofluorescence using Dmc1 and gamma-H2AX; however, the amount of DNA damage they observe in sycp2 mutants appears to be lower than that of wild-type, but not entirely eliminated (e.g. images in Figure 6A-iv and 6B-iv for Dmc1). The extent of DNA damage (via number of Dmc1 foci) in wild-type, sycp2, and spo11 mutants should be quantified to indicate the exact severity of the mutant phenotypes relative to each other and wild-type.

––– We added the quantification of Dmc1 signals in Fig 6B. Because many Dmc1 foci cluster around the telomere bouquet in zebrafish spermatocytes, counting numbers of discrete Dmc1 foci is difficult. Thus, the Dmc1-stained area was measured and compared among wild-type, *spo11* and *sycp2* mutant spermatocytes.

2. Using quantification of telomere foci in fixed cells, the authors suggest that chromosome pairing at the telomeres is partially impaired in the sycp2 hypomorph. As mutants for various meiotic chromosome structures have been shown in several systems to lead to nonhomologous pairing, the authors should definitively demonstrate whether homologous pairing is occurring at all in this mutant by using fluorescence in situ hybridization targeting an autosomal locus, as performed in a recent PLoS Genetics publication (Blokhina et al 2019). This result would provide important, strong validation of their conclusion and eliminate the possibility that nonhomologous pairing is occurring in this mutant, as occurs in chromosome structure mutants in other systems.

––– We evaluated homologous pairing by BAC in situ hybridization as performed in Blokhina et al. (PLOS Genet. 2019). These results are shown in Fig 5.

3. In the present study, the authors find an effect on meiotic recombination initiation by looking at Dmc1 staining in sycp2 mutants. Previous studies by Doug Bishop’s lab and Akira Shinohara’s lab have established that in S. cerevisiae, Rad51 is required for Dmc1 foci formation. To both further validate the finding of this manuscript that recombination initiation is inhibited and add additional mechanism to the action of Sycp2 in the early stages of meiotic recombination, the authors can evaluate Rad51 foci localization (using the Rad51 antibody from Blokhina et al., PLoS Genetics 2019) together with Dmc1 localization in wild type, spo11, and sycp2 mutants. Further, this co-localization experiment would be useful to determine whether Dmc1 and Rad51 colocalize (and the frequency of their colocalization) in zebrafish.

––– A recent report by Shi et al (PLOS Genet. 2019) showed that RPA is required for both RAD51 and DMC1 focus formation in mice. If this is also the case in zebrafish, not only Dmc1 but also Rad51 foci are unlikely to be formed in our *sycp2* knockout fish, in which Rpa foci were barely detected (Fig 7A).

Differential roles of Rad51 and Dmc1 are very interesting; however, our anti-Dmc1 antiserum would not be the best material to address this point. Our antiserum was raised against a part of the Dmc1 protein that is 43% identical to the zebrafish Rad51 protein (this information was added to the Materials and Methods). Thus, our anti-Dmc1 antiserum also potentially recognizes Rad51. Since, it is difficult to differentiate Dmc1 and Rad51 localizations with our anti-Dmc1 antibody, we could not pursue this point in our paper, unfortunately.

4. In the discussion, the authors draw comparisons between Sycp2 homologs in mice, yeast, nematodes, humans, and zebrafish. Supplementing this discussion with a specific bioinformatic analysis of conserved domains and sequence of zebrafish Sycp2 and Red1 (as was performed for the C-terminus of Red1 and mouse Sycp2 in West et al., eLife 2019) would strengthen their conclusion as well as possibly provide interesting insights into the components of synaptonemal axis proteins required for performing diverse roles in meiotic prophase I.

––– We added domain structures of mouse, zebrafish and budding yeast SYCP2/Red1 in S12 Fig. A. A sequence alignment of putative HORMAD/Hop1-binding domains from several species was also added to S12 Fig. B.

5. For Figure 3B, please indicate in both the image and figure legend what part of the image the inset panel is for the leptotene images.

––– In *sycp2^-/-^* and most *sycp2^its/its^* spermatocytes, the leptotene stage cannot be identified because of the lack of Sycp2 and Sycp3 filaments. According to Sycp1 appearances, those cells were at zygotene-like stages (i-iv). Some *sycp2^its/its^* spermatocytes showed pachytene-like appearances (v). Those classifications were added in the images and figure legend of Figure 3B, as well as those of other figures.

6. For Figure 5B, several changes would increase the clarity of this figure. First, please include the genotypes and stages of the images in the Figure itself. Second, the labels for the three- and four-color images are confusing, especially the label for Sycp3 (perhaps put the labels, with Sycp3 in white, all against a black background to clearly indicate that Sycp3 is in white in the images). Third, include better zoom-in panels of these images similar to Blokhina et al., PLoS Genetics 2019, they are zoomed in at a higher level with clear colors that enable the reader to see individual DSB sites along the axis near the telomeres.

––– Fig 5B was rearranged with new colors and magnification, as shown in Fig S8. This panel was moved from the main figures to the supplementary data, since DSB foci near telomeres have already been reported in Blokhina et al., PLoS Genetics 2019.

7. The ladder for the RT-qPCR in Figure S4B appears to be from a different gel than the result of the lanes in the gel; however, there is no indication either the presentation of the image or the figure legend that this is the case. This panel as well as the figure legend should be amended appropriately.

––– In both S4 Fig B and C, the left part or panel is a colorimetric image of the protein ladder on the same membrane. We added this information to the S4 Fig legend.

8. The western blot in Figure S4C appears very overexposed as well as extremely adjusted with post-processing methods outside of the dynamic range. Inclusion of panels from under/over exposed images of the blot as well as without any image adjustment should be included for this figure.

––– The blot image of S4 Fig C was replaced with the original raw data.

9. To improve clarity of the figure, Figure S5 should have the different meiotic stages indicated for the images.

––– S5 Fig A in the previous manuscript was rearranged to S5 Fig B in the new manuscript, with images of wild-type nuclei at different meiotic stages. S5 Fig B in the previous manuscript was moved to S6 Fig and is shown with all prophase I stages of wild-type spermatocytes that were rearranged from main Fig 4 in the previous manuscript.

10. In Figure S7, the DAPI levels for the spo11-/- mutant appears to be decreased significantly relative to the wild type images. Please check the exposures of the channel for these images.

––– We confirmed that all pictures with the same channel in this panel were taken at the same exposure. The difference in the DAPI signal intensity might come from staining, although all slides were processed at the same time. To avoid confusion, we replaced pictures with nuclei that have relatively similar DAPI intensities (S9 Fig B in the new manuscript).

11. Several of the findings of the authors have already been found in an earlier published study (Blokhina et al., PLoS Genetics 2019). For example, the authors include in their abstract that they demonstrate SC assembly occurs in the vicinity of telomeric regions (p. 1 line 12) and that DSB formation occurs adjacent to chromosome ends (p. 4 lines 87-88). As these results has already been shown by Blokhina et al., PLoS Genetics 2019, I suggest the authors remove these statements in the abstract and last paragraph of the intro where they present them as novel findings, and instead add stronger definition/emphasis of their own novel results from those results that were also found in the recent Burgess lab study.

––– We modified the abstract and the last paragraph of introduction and removed those statements.
