# Supplementary material for: Sycp2 is essential for synaptonemal complex assembly, early meiotic recombination and homologous pairing in zebrafish spermatocytes
Source: PLoS Genet. 2020 Feb 24;16(2):e1008640. doi: 10.1371/journal.pgen.1008640 (PMC7062287; doi:10.1371/journal.pgen.1008640)
Supplement: Attachment — Submitted filename: Comments_to_the_Reviewers_200122.docx [file pgen.1008640.s021.docx]

**Reviewer’s comments and author responses**

The authors appreciate the reviewers’ critical evaluation of the manuscript and helpful suggestions for improving it. As indicated in the responses that follow, reviewer comments and suggestions have been taken into account during the preparation of the manuscript revision.

Reviewer #1: The authors did a wonderful job addressing the comments and making the story richer with more data. Overall is a very nice paper.

Minor comments:

The first section of the discussion on the hypomorphic allele of sycp2its is redundant with the results. I think it can be left out completely since much of the discussion is already in the results section. It takes away from the important meiotic phenotypes the authors want to highlight.

–––––– We removed the first section of the discussion. Accordingly, we inserted the following sentence in the S4 Fig legend:

"The Sycp2 protein was not detected in *sycp2^its/its^* testes. However, we cannot exclude the possibility that there is expression of a truncated Sycp2 protein that is not recognized by our anti-Sycp2 antibodies specific to a C-terminal region of Sycp2."

Seeing 3 and 4 BAC probe spots in the sycp2 mutant indicates there may be a sister chromatid cohesion defect as the authors say. The foci are really far away from each other. Do you think the sister chromatids are completely separated from each other since there is not axis for them to anchor? It will take more experiments with multiple BAC probes to figure this out, but I was surprised. What does an sycp3 mouse mutant look like? Is that informative?

––––––We appreciate this comment. In mice, no *Sycp3^-/-^* spermatocytes with >40 CREST foci are observed (Yuan et al, 2000), suggesting that sister chromatid cohesion at the centromeric regions is not affected in the absence of SYCP3. This is in contrast to our observations in *sycp2* mutant zebrafish in which >100 telomere foci and >2 BAC foci in some spermatocytes. This difference made us re-analyze the BAC experiment data again, by taking the distances of BAC spots into account. Actually, an associated locus can be observed as two close BAC foci when the BAC-targeted locus is located on a loop region (i.e. Ishiguro et al, 2014). The results showed that two spots among them were located nearby in most cases, and nuclei in which 3-4 spots were scattered were rare. Two adjacent spots may reflect the distance of chromatin loops of sister chromatid. We realized that the cohesion defect descriptions were over-speculation in the present data. We changed Figure 5 and removed descriptions about cohesion defects.

Reviewer #2: Re-review: Sycp2 is essential for synaptonemal complex assembly, early meiotic recombination
and homologous pairing in zebrafish spermatocytes

Overall manuscript is much tighter and easier to read and the authors have addressed a number of the issues raised by the reviewers.

1) The main issue they do NOT address is the role of the checkpoint in the loss of female embryos. In multiple places they said “in the interest of time” However, the paper was submitted initially in July and returned. If they had done the experiment it would have delayed another 3 months, true, but this is one of the novel aspects of the work and would indeed answer a critical question.

In the description of this arrest, the text is unclear for non-zebrafish readers. On p.8, in the observation that there are no female offspring and that it is required for ovarian development, the link between ovarian development and sex determination needs to be better described. One suggested is to add the text that was provided as a response to the reviewers:

“We speculate that the checkpoint is rather active in both oogenesis and
spermatogenesis in sycp2-/- zebrafish. In zebrafish, all juveniles develop gonads with
immature oocytes regardless of their definitive sex. The individual in which these
immature oocytes degenerate becomes male. Therefore, zebrafish mutants depleted of
oocytes develop as males that are mostly infertile. In sycp2-/- juvenile gonads, we
observed previtellogenic dictyate oocytes in four among five individual fish (S10 Fig).
Therefore, the absence of females in sycp2 mutant fish (Table 1) implies that oocytes
were eliminated at the juvenile age, possibly due to checkpoint activation in oogenesis.
As a result, all sycp2 mutant fish develop as males.”

–––– We appreciated the suggestion and modified the 3rd section of Result on p.8.

2) In response to comment #4 about distinguishing whether the lack of DMC1 foci is a defect in DSBs or early DSB processing, the authors claim they did not have time to find the right dose of IR. This is actually a poor excuse: if the endpoint is sterility, this is an issue, but they could easily have looked for RAD51 and DMC1 foci within 30 minutes of irradiation to check whether these were detectable. This is still a do-able and important experiment.

–––– Irradiation experiments are not easy in Japan because the use of radiation is strictly regulated by law. Many Universities don’t have radiation source for this kind of experiments, and our institute does not. Therefore, we need to match the experiment with the schedule of staffs of another University. At the first revision, we have tried to analyze the Dmc1/Rad51 foci, but we could not obtain enough data to give a conclusion. At this time, with help of Dr. Carlton at Kyoto Univ, we tried it again and obtained them. We added these results as S11 Fig.

Text:

Rpa foci should be RPA foci.

–––– Rpa in the main text, figure legends and Fig. 7A were modified to RPA.

Line 362: I would say that in the fanc1 and brca2 mutants that they became “sterile males”

–––– In the line 362, we changed "males" to "sterile males".

Reviewer #3: The authors have made a good effort to address reviewer concerns. Their work is strengthened by their removal and deemphasis of their identical results to that already published in Blokhina et al PLoS Genetics 2019. Further, their addition of data in Figure 5 to demonstrate pairing defects in the sycp2 mutant alleles also strengthens the paper. It is unfortunate that many of the comments of all three reviewers were unable to be addressed due to lack of time. The addition of several of the requested experiments, such as assessing rescue of DSBs and fertility by irradiation of sycp2 mutants. With regards to some of their responses to reviewers, I have two major comments/concerns that decrease the impact of their work and strength of their conclusions:

––––– As mentioned in a response to the Reviewer 2, we added results of DSB rescue experiment in S11 Fig.

1) The authors mention in their response to Reviewer#3 comment #3 that their Dmc1 “antiserum was raised against a part of the Dmc1 protein that is 43% identical to the zebrafish Rad51. Thus, our anti-Dmc1 antiserum also potentially recognized Rad51.” This fact regarding their Dmc1 antibody is troubling because it may not be specific to Dmc1. The authors should either 1) amend their discussion of these results and their figures to reflect the fact their Dmc1 antibody may actually be a Dmc1/Rad51 antibody (all reference to Dmc1 should be changed to Dmc1/Rad51); or, 2) they should directly determine whether their antibody is specific to Dmc1 by looking at localization in a dmc1 null mutant by IF and/or Western.

­­­­­––––– We corrected all "Dmc1 foci" to "Dmc1/Rad51 foci". In addition, we inserted the following sentence in Result (line 293):

"It should be noted that our anti-Dmc1 antiserum potentially recognizes another recombinase, Rad51, since they share high similarity at the amino acid sequence level (see Materials and Methods)."

2) In response to Reviewer #1 comment #5, the authors look at Rad21l1l localization with Sycp1 stretch to determine whether the SC is forming between sisters. Although they see colocalization of Rad21l1l and Scyp1, this is not a definitive determinant of whether Sycp1 is forming between sisters because Sycp1 could be forming along a single sister chromatid axis. In order to determine if Sycp1 is forming between sisters, the two sister chromatid axes need to be resolved and Sycp1 needs to be see forming between those two axes. The use of DeltaVision deconvolution microscopy is not at a sufficient resolution level to resolve two axes. The authors need to use SIM microscopy or EM to determine the formation of SC between two sister chromatid axes (as was done in Cahoon CK, Helm JM, and Libuda DE Genetics 2019 and Xu H, Beasley MD, … McKay MJ Dev Cell 2005 respectively). Alternatively, the authors could remove this data and simply postulate in the discussion that Sycp1 forming between sister chromatids in this context.

––––– We agree that our Deltavision images don't have enough resolution to determine whether aberrant Sycp1 fragments in *sycp2* mutant spermatocytes were formed between sister chromatids. That was mis-leading. However, this data can indicate that Sycp1 fragments were not formed anywhere else but were loaded onto chromatin. Therefore, we kept this data as a supplementary figure (S8 Fig), and replaced "cohesin axes" with "chromatin" in line 250 in Result. We also modified Discussion and added as following:

"We also observed costaining of aberrant Sycp1 filaments in *sycp2-/-* spermatocytes with stretches of Rad21l1 that seem to exist, at least in part, even in the absence of Sycp2 (S8 Fig). It was difficult to determine whether these Sycp1 filaments were loaded between sister chromatids, at our image resolution. In the case that the aberrant Sycp1 filaments were formed between sister chromatids, as observed in a hypomorphic *Stag3* mutant mouse, in which the levels of the cohesin subunit REC8 are partly reduced (43), Sycp2 might function to prevent formation of the synaptonemal complex between sister chromatids."
